# Water permeability of the mammalian cochlea: functional features of an aquaporin-facilitated water shunt at the perilymph–endolymph barrier

**DOI:** 10.1007/s00424-013-1421-y

**Published:** 2014-01-03

**Authors:** A. Eckhard, M. Müller, A. Salt, J. Smolders, H. Rask-Andersen, H. Löwenheim

**Affiliations:** 1 Hearing Research Center, Department of Otorhinolaryngology—Head & Neck Surgery, University of Tübingen Medical Centre, Elfriede-Aulhorn-Strasse 5, 72076 Tübingen, Germany; 2 Department of Otolaryngology, Washington University School of Medicine, St. Louis, MO USA; 3 Department of Physiology II, Goethe-University, Frankfurt am Main, Germany; 4 Department of Surgical Sciences, Section of Otolaryngology, Uppsala University Hospital, Uppsala, Sweden

**Keywords:** Aquaporin, Cochlea, Endolymph, Perilymph, Water permeability, Ménière's disease

## Abstract

The cochlear duct epithelium (CDE) constitutes a tight barrier that effectively separates the inner ear fluids, endolymph and perilymph, thereby maintaining distinct ionic and osmotic gradients that are essential for auditory function. However, in vivo experiments have demonstrated that the CDE allows for rapid water exchange between fluid compartments. The molecular mechanism governing water permeation across the CDE remains elusive. We computationally determined the diffusional (*P*_D_) and osmotic (*P*_f_) water permeability coefficients for the mammalian CDE based on in silico simulations of cochlear water dynamics integrating previously derived in vivo experimental data on fluid flow with expression sites of molecular water channels (aquaporins, AQPs). The *P*_D_ of the entire CDE (*P*_D_ = 8.18 × 10^−5^ cm s^−1^) and its individual partitions including Reissner's membrane (*P*_D_ = 12.06 × 10^−5^ cm s^−1^) and the organ of Corti (*P*_D_ = 10.2 × 10^−5^ cm s^−1^) were similar to other epithelia with AQP-facilitated water permeation. The *P*_f_ of the CDE (*P*_f_ = 6.15 × 10^−4^ cm s^−1^) was also in the range of other epithelia while an exceptionally high *P*_f_ was determined for an epithelial subdomain of outer sulcus cells in the cochlear apex co-expressing AQP4 and AQP5 (OSCs; *P*_f_ = 156.90 × 10^−3^ cm s^−1^). The *P*_f_/*P*_D_ ratios of the CDE (*P*_f_/*P*_D_ = 7.52) and OSCs (*P*_f_/*P*_D_ = 242.02) indicate an aqueous pore-facilitated water exchange and reveal a high-transfer region or “water shunt” in the cochlear apex. This “water shunt” explains experimentally determined phenomena of endolymphatic longitudinal flow towards the cochlear apex. The water permeability coefficients of the CDE emphasise the physiological and pathophysiological relevance of water dynamics in the cochlea in particular for endolymphatic hydrops and Ménière's disease.

## Introduction

The inner ear is a fluid-filled sensory organ enclosing two unique extracellular fluids, perilymph and endolymph. One of the most fundamental questions regarding inner ear function is how fluid regulation maintains the delicate balance of ion gradients and fluid volume between the perilymph and endolymph.

**Fig. 1:**
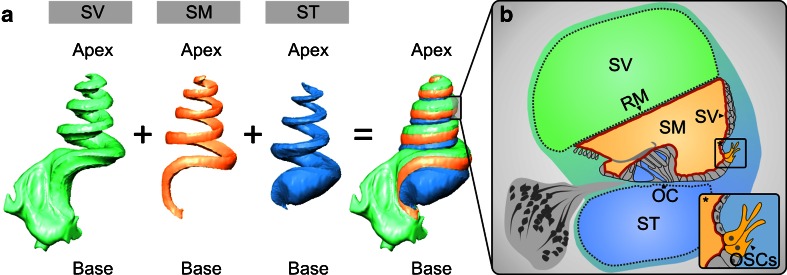
Three-dimensional (3D) reconstruction of orthogonal-plane fluorescence optical sectioning (OPFOS) data from the guinea pig cochlea to demonstrate the anatomical relations of the cochlear fluid spaces. **a** 3D reconstruction of the endolymphatic space in the scala media (*SM*) and the perilymphatic spaces in the scala tympani (*ST*) and scala vestibuli (*SV*). **b** Schematic cross-sectional view of the guinea pig cochlear duct in the half-turn V. The cochlear duct epithelium and interepithelial tight junctions constitute the cochlear “perilymph–endolymph barrier” (PEB, *red line*) that encloses the endolymph in the SM. Two partitions of the cochlear PEB, namely Reissner's membrane (*RM*) and the organ of Corti (*OC*), directly separate the endolymph in the SM from the perilymph in the SV and ST. The stria vascularis (*SV*) does not form a direct epithelial barrier between the cochlear fluid compartments of SV, SM and ST. In the inlay *, the position of the outer sulcus cells (OSCs) in the cochlear duct epithelium is illustrated.

In the cochlea, the endolymph in the scala media (SM) is separated from the perilymph in the scala vestibuli (SV) by Reissner's membrane (RM) and from the perilymph in the scala tympani (ST) by the epithelium residing on the basilar membrane that includes the organ of Corti (OC) and the epithelial lining of the inner and outer sulcus (Fig. 1a, b). Laterally, the epithelial lining of the spiral ligament (SL) including the stria vascularis closes the cochlear duct. The entire cochlear duct epithelium (CDE) is sealed by intercellular tight junctions and thereby forms the cochlear “perilymph–endolymph barrier” (PEB; [36]; Fig. 1b).

The rate constants (*P*′) for the perilymphatic–endolymphatic exchange of potassium (K^+^, *P*′ = 112.29 × 10^−6^ s^−1^), sodium (Na^+^, *P*′ = 6.37 × 10^−6^ s^−1^), chloride (Cl^−^, *P*′ = 22.58 × 10^−6^ s^−1^) and water (H_2_O, *P*′ = 15 × 10^−3^ s^−1^) demonstrate a high permeability for both ions and water for the entire CDE [43–45]. The related turnover half-time of the CDE for potassium is 55 min [45], while that for water is only ~8 min [78]. At the molecular level, several transmembrane proteins have been identified in the CDE that specifically facilitate the transepithelial exchange of K^+^, Na^+^ and Cl^−^, consistent with the high electrolyte permeability of the cochlear PEB [reviewed in 51]). Although *P*′ for water exchange across the cochlear PEB is 130 times greater than that for K^+^, surprisingly molecular pathways that specifically facilitate water permeation across the CDE have not been elucidated. As water is known to diffuse with low permeability through lipid bilayer membranes, the major determinant of membrane water permeability in many physiological processes is the presence or absence of molecular water channels, notably aquaporins (AQPs) [6, 42, 95]. The expression of eight AQP subtypes has been confirmed in the heterogeneous cell population of the CDE and its surrounding connective tissue, including AQP1–AQP7 and AQP9 [reviewed in 17]). Commonly used parameters that provide a quantitative measure of water exchange across epithelia and describe the nature of transepithelial water exchange (i.e., solubility–diffusion through the lipid bilayer membrane or aqueous pore (e.g., AQP)-facilitated water permeation) are the diffusional (*P*_D_) and osmotic (*P*_f_) water permeability coefficients. *P*_D_ is a measure of the rate of water exchange across an interface (per unit area) based on thermal movements in the absence of an osmotic or hydrostatic gradient. *P*_f_ describes overall water movement (per unit area) as a response to hydrostatic or osmotic pressure gradients. As a quantitative and comparative measure, *P*_D_ and *P*_f_ have been determined for various AQP-expressing epithelia (reviewed in [96]); however, despite the established, abundant expression of AQPs in the CDE, *P*_D_ and *P*_f_ have not been established for this epithelium, and the functional significance of AQPs in transepithelial water exchange between the cochlear perilymph and endolymph remains unknown.

In this study, we examined the hypothesis that water homeostasis in the cochlear perilymph and endolymph is maintained by AQP-based transepithelial water permeation. To this end, we determined *P*_D_ and *P*_f_ for the entire CDE and for its individual partitions, including RM, the OC and a particular epithelial subdomain in the cochlear apex comprised of a subpopulation of outer sulcus cells (OSCs; inlay * in Fig. 1b). Notably, in this subpopulation of OSCs in the rat and human cochlea, AQP4 was localised in the basolateral membrane, which stretches into the perilymphatic fluid of the spiral ligament, and AQP5 was localised in the apical membrane, which is bathed in endolymph in the SM [31]. This localisation of two AQPs in both cellular membrane domains (herein referred to as “complementary” membranous AQP expression) constitutes the molecular and cellular basis of an AQP-facilitated “water shunt” between the perilymph and endolymph across the PEB in the cochlear apex [18, 31]. To date, this subpopulation of OSCs in the cochlear apex constitutes the only confirmed cell type in the CDE exhibiting a complementary membranous AQP expression.

The calculations of *P*_D_ and *P*_f_ in this study were based on previously derived in vivo experimental data on diffusional [44] and osmotic [78] water exchange between the perilymphatic and endolymphatic fluid compartments to respectively determine *P*_D_ and *P*_f_ for the entire CDE, its individual partitions including RM and OC as well as the epithelial subdomain in the cochlear apex comprised by the subpopulation of OSCs co-expressing AQP4 and AQP5.

Furthermore, cochlear water dynamics simulations were performed using a modified version of the *Cochlear Fluids Simulator* V. 1.6i [79]. The applicability of this computer model to the simulation of water exchange between cochlear–fluid compartments of the guinea pig cochlea was validated by comparing the in silico (i.e. via computer simulations)-generated data with the in vivo data determined on guinea pig cochleae in a study by Konishi et al. [44]. Accordingly, the diffusional water exchange was simulated to determine *P*_D_ across the entire CDE that is between the perilymph of SV and ST and the endolymph of SM (SV + ST/SM model) as well as its individual partitions which is between the SV and SM (across RM; SV/SM model) and between the ST and SM (across OC; ST/SM model).

As *P*_D_ and *P*_f_ are defined as the transport flux of water per unit membrane area [19], surface areas of the entire CDE, RM and the OC were quantified from histological sections and previously derived morphological data of cochlear fluid space dimensions from the adult guinea pig cochlea [32]. The membrane area of complementary AQP expression in OSCs of the cochlear apex was determined by measurements of immunohistochemically labelled sections of the adult guinea pig cochlea.

We established values of *P*_D_ and *P*_f_ for the entire CDE of the guinea pig cochlea and its individual partitions (RM, OC and OSCs in the cochlear apex) that indicate aqueous pore-facilitated water permeation. The abundant AQP expression in the CDE provides a plausible molecular basis for rapid perilymphatic–endolymphatic water exchange. For the epithelial domain of AQP4/AQP5-expressing OSCs in the cochlear apex, we determined an exceptionally high *P*_f_ that is comparable to the *P*_f_ values reported for renal tubule epithelia. Furthermore, we present a new model of longitudinal endolymph flow that incorporates a perilymphatic–endolymphatic water exchange across a high-transfer AQP-facilitated “water shunt” in the cochlear apex. Based on this model, we provide a molecular explanation for experimentally determined phenomena of endolymphatic longitudinal flow towards the cochlear apex in the dehydrated cochlea.

## Materials and methods

### Animals

Adult male, pigmented guinea pigs (strain BFA bunt) weighing 700–800 g were obtained from an in-house breeding colony. The animals were maintained in an in-house animal facility with free access to food and water under standard white cyclic lighting. Three cochleae from three different animals were cryosectioned and used for double-immunolabelling of the water channel proteins AQP4 and AQP5 (*n* = 3, Fig. 5b) to determine the radial length of the apical membranes of OSCs that exhibited complementary membrane localisation of AQP4 and AQP5. The remaining three cochleae were used for whole-mount preparations of the lateral wall of the cochlear duct (*n* = 3, Fig. 5c, d). The whole-mount preparations were double-immunolabelled for AQP4 and AQP5 to determine the longitudinal length of complementary AQP4/AQP5 localisation in OSCs. Azan-stained sections from two adult guinea pig cochleae, obtained from the histology collection of the Institute of Anatomy of the University of Tübingen, were used to measure the radial length of RM (PEB between the SV and SM) and the OC, which in this study comprises the epithelial lining on the basilar membrane reaching from the inner to the outer sulcus (PEB between the ST and SM; *n* = 2, Fig. 4a, c).

### Inner ear dissection, fixation, decalcification, embedding, sectioning and whole-mount preparation

The animals were deeply anesthetised by intraperitoneal injection of a mixture of fentanyl (0.025 mg kg^−1^; Albrecht GmbH, Aulendorf, Germany), midazolam (1.0 mg kg^−1^; Ratiopharm, Ulm, Germany) and medetomidine (0.2 mg kg^−1^; Albrecht GmbH), and sacrificed with an intrapulmonary injection of embutramide (0.5 ml; T61, Intervet, Unterschleissheim, Germany). Subsequently, transcardial perfusion with a warm (~37 °C) 0.9 % sodium chloride solution was conducted (~100 ml) and followed by perfusion with warm (~37 °C) 4 % paraformaldehyde (PFA) (Carl Roth GmbH, Karlsruhe, Germany) in phosphate-buffered saline (PBS; ~400 ml) until fixation-induced stiffness of the neck was confirmed. The brain was removed, and dissection of the complete bony labyrinth capsules from the skull base was conducted in ice-cold 4 % PFA in PBS. The perilymphatic spaces of each cochlea were opened by removing the stapedial footplate from the oval window niche and removing the round window membrane. Fixation of the inner ear was performed via a gentle perfusion of the opened perilymphatic scalae with 4 % PFA, followed by a 2-h immersion in 4 % PFA. The bony capsules of the cochleae were thinned using a high-speed motorised drill prior to decalcification of the fixed specimens for 48 h in 2 mM EDTA in PBS. For cryosectioning, the cochleae were immersed in 25 % sucrose in PBS overnight and embedded in a cryo-gel (Tissue-Tec® O.C.T. compound, Sakura Finetek, Zoeterwoude, Netherlands). Midmodiolar cryosections intended for immunolabelling were cut at 14 μm lateral thickness using a cryostat (Microm, Thermo Fisher Scientific, Walldorf, Germany) and were immediately mounted on SuperFrost® Plus microscope slides (Langenbrinck, Emmendingen, Germany). For lateral wall whole-mount preparations, the otic capsule was carefully removed, and the lateral wall was then separated from the OC in the region of the Claudius cells (CCs) along the entire length of the cochlear duct and divided into 8 to 11 segments; these segments were used for immunolabelling.

### Immunofluorescence labelling of aquaporin-4 and aquaporin-5

Based on a previous study demonstrating the complementary membranous expression of AQP4 and AQP5 in OSCs in the rat cochlea [31], AQP4 and AQP5 immunolabelling in the guinea pig cochlea was performed using a polyclonal goat anti-AQP4 antibody (Santa Cruz Biotechnology Inc., Santa Cruz, CA, USA; dilution 1:400) and a polyclonal rabbit anti-AQP5 antibody (Millipore, Billerica, MA, USA; dilution 1:100), visualised with an Alexa 594-conjugated anti-goat secondary antibody (Molecular Probes–Invitrogen, Carlsbad, CA, USA; dilution 1:400) and an Alexa 488-conjugated anti-rabbit secondary antibody, both of which had been raised in donkey (Molecular Probes–Invitrogen; dilution 1:400). All antibodies were diluted in PBS supplemented with 0.1 % Triton-X 100 and 0.5 % NDS. Lateral wall whole-mount preparations were stained during free-floating incubation. All cryosections and lateral wall whole-mount preparations were coverslipped using FluorSave™ mounting medium (Calbiochem-Merck, Darmstadt, Germany).

### Microscopic analysis

Azan-stained sections were photographed using a Zeiss Axioplan 2 microscope (Zeiss, Göttingen, Germany). Immunolabelled cryosections and whole-mount preparations were analysed using a Zeiss 510 Meta laser-scanning microscope (Zeiss) and a Zeiss Axioplan 2 microscope (Zeiss), respectively.

### Length measurements of the cochlear perilymph–endolymph barrier

The radial length (width) of RM (the membrane separating the perilymph in SV from the endolymph in SM) and the width of the OC (separating the perilymph in ST and the endolymph in SM) were determined in each of the eight cochlear half-turns (I–VIII, excluding the hook region) on midmodiolar azan-stained sections of the adult guinea pig cochlea, which were derived from two different animals (*n* = 2). The width of RM was determined between its two insertion points (at the spiral limbus and the spiral ligament). The width of the OC was measured along the apical cell surfaces, extending from the inner sulcus cells of the spiral limbus to the OSCs of the spiral ligament. The surface of the stria vascularis that constitutes the third epithelial portion of the cochlear PEB was not measured. We did not include the stria vascularis in our computational model because it does not provide a direct interface between the perilymphatic spaces of SV and ST and the endolymph in SM. Furthermore, water movement across the lateral wall (and the stria vascularis) does not seem to play an important role for perilymphatic–endolymphatic water exchange, as was suggested by Konishi et al. [44]. Measurements were performed using AxioVision software (V 4.8.2.0, Zeiss).

Data on the baso-apical length of the cochlear spiral of the adult guinea pig were derived from Hofman et al. [32] and used to determine the surface area of RM and the OC. In their original description, Hofman et al. [32] described the length of full cochlear turns, but not of each cochlear half-turn. Therefore, an XY projection of the cochlear spiral derived from 3D reconstruction data of the guinea pig cochlea was used to measure the individual longitudinal lengths of the eight cochlear half-turns and the hook region. Measurements were performed using the software ImageJ (V. 1.42q; National Institutes of Health, Bethesda, MD, USA).

### Length measurements of complementary AQP4 and AQP5 membrane localisation in OSCs

The radial width of the apical membrane of OSCs that exhibit complementary localisation of AQP4 and AQP5 in their basolateral and apical membrane domains was determined by length measurements on immunolabelled cryosections of the adult guinea pig cochlea, which were derived from three different animals (*n* = 3). Corresponding longitudinal length measurements of AQP4 and AQP5 expression in the baso-apical direction were also made in OSCs from immunolabelled whole-mount preparations of the cochlear duct lateral walls derived from the remaining three cochleae of the same three animals (*n* = 3). The baso-apical length of AQP4 expression was determined in CCs and OSCs; the baso-apical length of AQP5 expression was measured in OSCs. The software AxioVision (V 4.8.2.0, Zeiss) was employed for the length measurements.

### Data on diffusional and osmotic water exchange between the cochlear fluid compartments

**Fig. 2:**
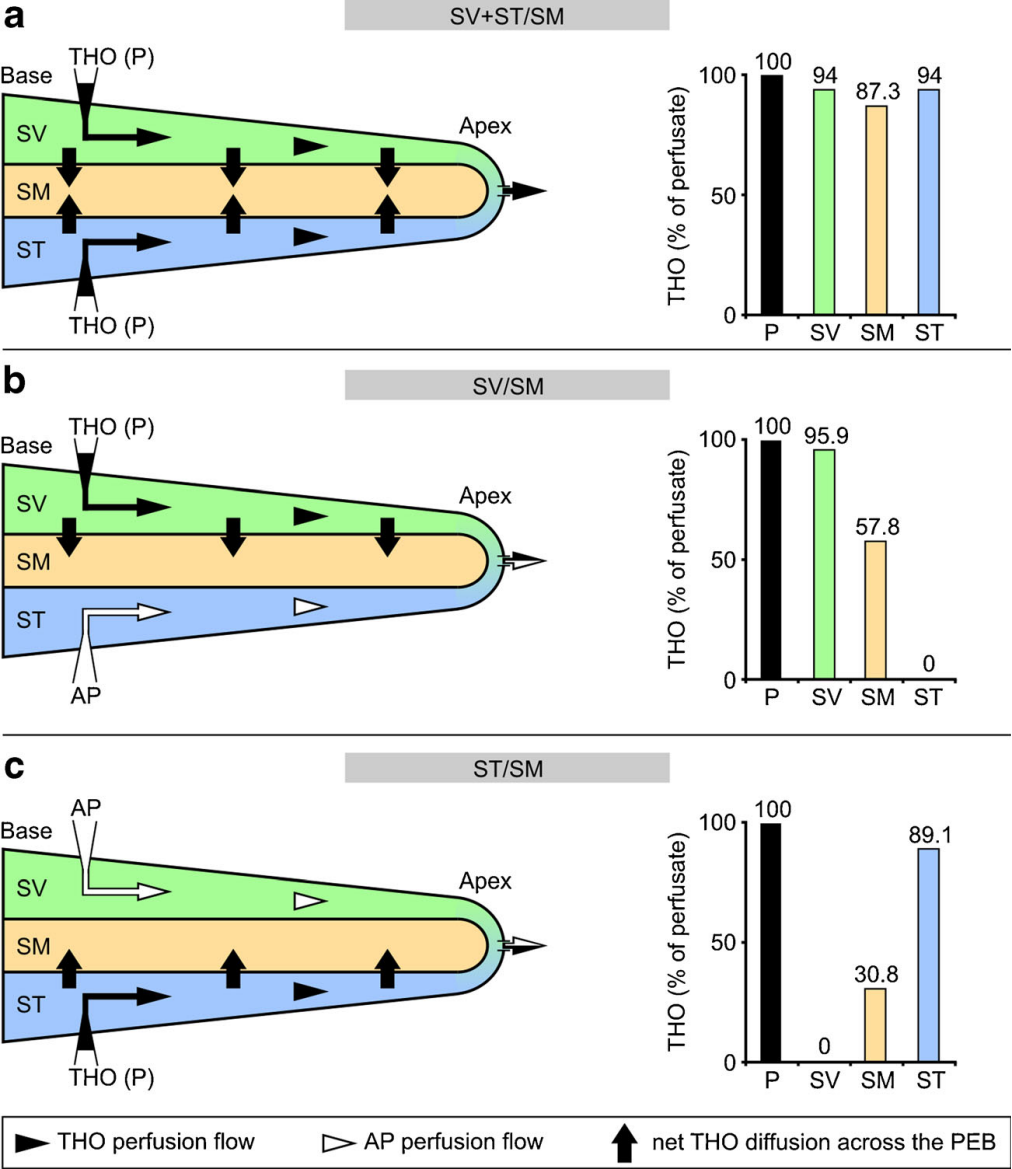
Experimental models for the determination of diffusional perilymphatic–endolymphatic water exchange in the in vivo experimental study by Konishi et al. [44]. **a** In the SV + ST/SM model, both perilymphatic scalae, namely the scala vestibuli (*SV*) and the scala tympani (*ST*), of the adult guinea pig cochlea were perfused with a fluid (*P*) containing tritiated water (*THO*). A cochleostomy in the helicotrema region served as an outlet for the perfusion fluids. Transepithelial THO diffusion into the endolymphatic fluid compartment of the scala media (*SM*) occurred across the cochlear perilymph–endolymph barrier (PEB) between the SV and SM, as well as between the ST and SM. THO concentrations in the SV, ST and SM were measured 7 min after the initiation of perilymphatic perfusion (the diagram shows the relative THO concentrations in the perfusion fluid (100 %) and the cochlear). **b** In the SV/SM model, the SV was perfused with THO, while artificial perilymph (*AP*) was injected into the ST. THO diffused from the SV to the SM across the portion of the cochlear PEB that is interposed between these two fluid compartments (i.e. Reissner's membrane). **c** In the ST/SM model, the ST was perfused with THO and the SV was rinsed with AP. THO diffused from the ST to the SM across the portion of the cochlear PEB that separates the ST and SM (i.e. the organ of Corti). Adapted from [44] with permission from the publisher, *Elsevier*

Although several studies have investigated the dispersal of macromolecular marker substances, such as trypan blue [3, 75], fluorescein [22, 23], thorotrast [4] or peroxidase [37], between the endolymphatic and perilymphatic spaces of the inner ear in vivo, these techniques were not adequate to investigate permeation through the highly water-specific AQP channels. In contrast, radioactively labelled water (tritiated water; THO) has a similar molecular structure as the water molecule (H_2_O) with the exception of one hydrogen (H) that is substituted with tritium (^3^H). Because of this structural similarity, AQPs exhibit comparable permeability characteristics for H_2_O and THO as determined on erythrocyte membranes (reviewed in [7]) that contain AQP1 [73], AQP3 [74] and AQP5 water channels [1]. Hence, in this study, we used empirical data from the study by Konishi et al. that described the diffusional exchange of THO between the perilymphatic and endolymphatic spaces in in vivo experiments in the adult guinea pig cochlea [44]. In their study, Konishi et al. used the following three experimental setups to determine transepithelial diffusional THO dynamics in the cochlea: (1) they measured the THO concentration in SM during THO perfusion of SV and ST to determine the rate of diffusional water flow across the entire cochlear PEB (hereafter referred to as model SV + ST/SM, Fig. 2a); (2) they measured the THO concentration in SM during THO perfusion of SV only to determine the rate of diffusional water flow across the partition of the PEB that separates the SV and SM (hereafter referred to as model SV/SM, Fig. 2b); and (3) they measured the THO concentration in SM during THO perfusion of ST only to determine the diffusional water flow across the partition of the PEB that separates the ST and SM (hereafter referred to as model ST/SM, Fig. 2c). The THO concentrations in each of the scalae were measured in vivo by Konishi et al. [44] 7 min after the onset of perilymphatic THO perfusion. The results of these THO measurements are given in the diagrams in Fig. 2a–c. The experimental setup in the study by Konishi et al. [44] enabled the measurement of the initial endolymphatic THO concentration changes within the first 20 min after the onset of perilymphatic THO perfusion because of the 1-min time intervals between measurements, starting at 3 min after the onset of perilymphatic THO perfusion.

**Fig. 3:**
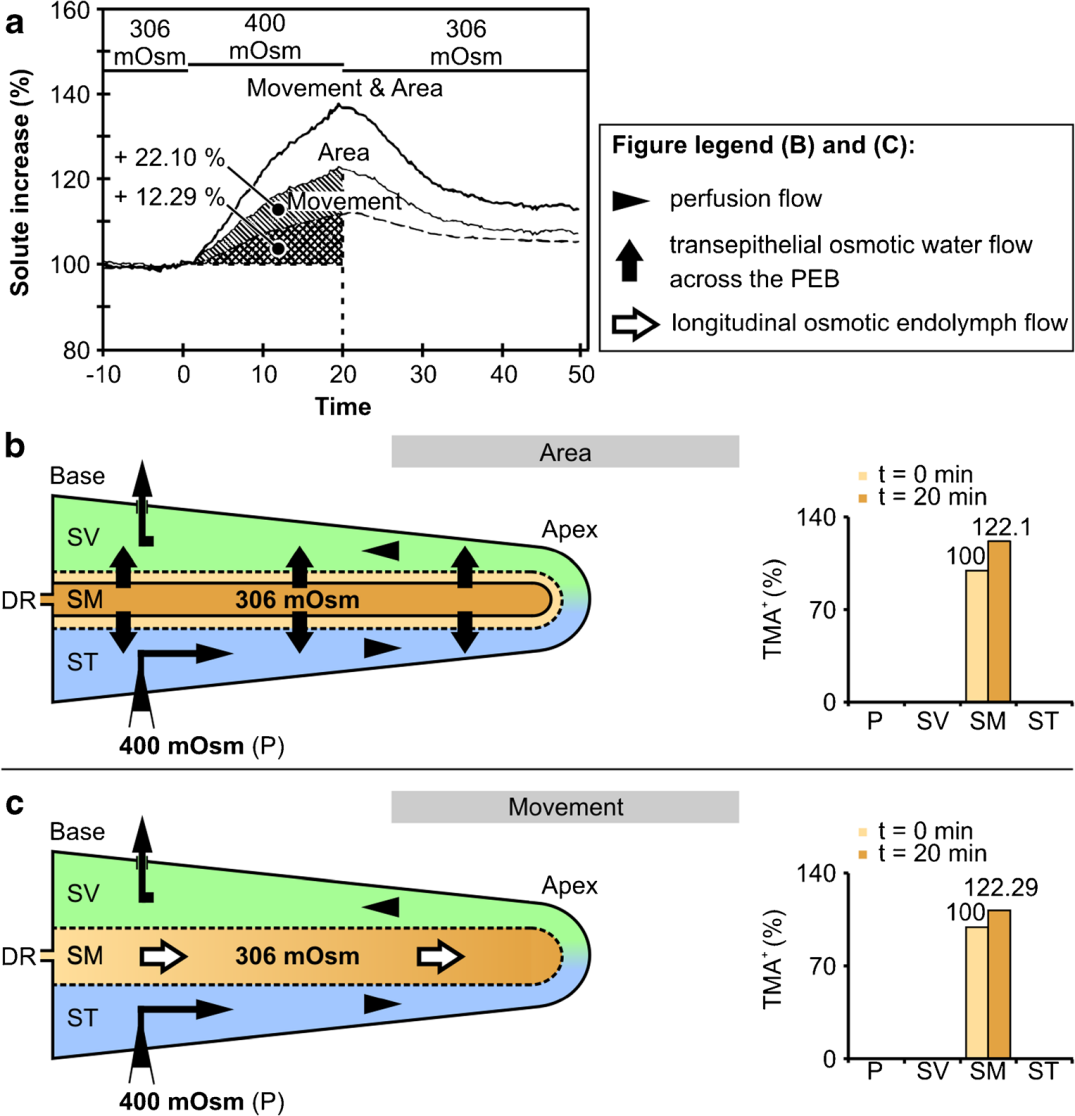
Mechanisms of endolymphatic volume marker increase during perilymphatic perfusion with hypertonic media in the in vivo experimental study of Salt and DeMott [78]. Hypertonic (400 mOsm (kg H_2_O)^−1^) perfusion of the perilymphatic scalae (scala vestibuli, *SV*; scala tympani, *ST*) in the adult guinea pig cochlea induced osmotic volume changes of the endolymph in the scala media (*SM*). These volume changes were quantified by Salt and DeMott [78] by measuring the concentration change of the ionic volume marker tetramethylammonium (TMA^+^) after its iontophoretic injection into the endolymph prior to hypertonic perilymphatic perfusion. Salt and DeMott identified two different mechanisms that accounted for the increase in endolymphatic TMA^+^, i.e. shrinkage of the endolymphatic compartment (**a** and **b**, area; TMA^+^ increased by 22.1 %) and apically directed longitudinal flow of TMA^+^-loaded endolymph (**a** and **c**, movement; TMA^+^ increased by 12.29 %). As the epithelial boundary of the cochlear duct is nearly impermeable to TMA^+^, the induced endolymphatic TMA^+^ increase can be attributed to a loss of endolymph volume that was proportional to the measured TMA^+^ increase. *DR*, ductus reunions; adapted from [78] with permission from the publisher, *Elsevier*

The osmotic water permeability coefficients (*P*_f_) of the cochlear PEB were also determined based on empirical data from the literature [78]. These data were derived from in vivo measurements of endolymphatic volume changes during perilymphatic perfusion with a solution that was hypertonic (400 mOsm (kg H_2_O)^−1^ [78]) compared with the isotonic endolymph (306 mOsm (kg H_2_O)^−1^ [78]). Hypertonic perilymphatic perfusion generated an osmotic pressure differential between the perilymph and endolymph that led to an outflow of water from the endolymphatic fluid compartment. This outflow was quantified by measuring the relative increase in the ionic volume marker tetramethylammonium (TMA^+^) in the endolymphatic fluid compartment with TMA^+^-sensitive electrodes [78]. As the cochlear duct epithelium surrounding the endolymphatic fluid compartment exhibits extremely low permeability to TMA^+^, the increase in the levels of this marker in the endolymph was approximately proportional to the water lost from the endolymphatic space during hypertonic perilymphatic perfusion. According to Salt and DeMott [78], the relative TMA^+^ increase in endolymph during hypertonic perilymphatic perfusion resulted from two different mechanisms: (1) reduction of the endolymph volume due to the flow of water from the endolymph into the perilymph (“Area”, endolymphatic TMA^+^ +22.10 %, Fig. 7a, b) and (2) an apically directed TMA^+^-loaded endolymph flow (“Movement”, endolymphatic TMA^+^ +12.29 %, Fig. 3a, c). As the TMA^+^ increase in the endolymph was caused by a proportional outflow of water from the endolymphatic compartment, we calculated osmotically induced transepithelial flows from the endolymph to perilymph (*J*_v_) to determine *P*_f_ for the entire cochlear PEB and for OSCs that exhibit complementary membranous expression of AQP4 and AQP5.

### In silico simulations of endolymphatic THO uptake during perilymphatic THO perfusion

Computer simulations of diffusional THO dispersal in the cochlear fluid compartments of SV, ST and SM were performed using the Washington University Cochlear Fluids Simulation Program (Cochlear Fluids Simulator, V. 1.6i), a freely accessible program available at http://oto2.wustl.edu/cochlea/ [79]. The program enables the simulation of the dispersal of drugs or other substances in the morphometrically modelled inner ear fluid spaces of different mammalian species (bat, chinchilla, gerbil guinea pig, mouse, rat and human) based on the combination of physical processes involved in solute dispersal: diffusion, longitudinal fluid flow and clearances to other compartments of the inner ear. In this study, we applied the fluid space dimensions of the guinea pig cochlea, since the in vivo data on cochlear diffusive water dynamics that we integrated in our model were derived from the guinea pig [44]. Using the Cochlear Fluids Simulator software, the diffusion coefficient of THO (2.3 × 10^−9^ m^2^ s^−1^) for the cochlear PEB was calculated based on the formula weight (22.03 mol^−1^). Other parameters used to configure the program were adapted from Konishi et al. [44] and are given in Table 1.

**Table 1:** In vivo experimental parameters in the study by Konishi et al. [44] and their adaption in the present study for in silico simulations using the Cochlear Fluids Simulator (V. 1.6im, modified)

	Konishi et al. [44]	Cochlear Fluids Simulator
Species	Guinea pig	Guinea pig
Tracer substance (TS)	THO	THO (MW: 22.0315)
TS-diff.-coeff.		2.3004 × 10^−5^ cm^2^ s^−1^
TS-conc. in perfusate	2 μCi ml^−1^ (normalised to 100 %)	100 %
TS-perfusion rate	8 μl min^−1^	8 μl min^−1^
TS-perfusion period	3–20 min	0–120 min
TS-entry site	Basal turn	Basal turn (0.1 mm from base)
TS-exit site	Helicotrema	Apical end of SV (15.5 mm from base) and ST (16.2 mm from base)
TS-method of conc. measurements	Endolymph sampling	Continuous measurements
Location of measurement	SM (basal turn)	SM, SV, ST (1 mm from base)
Scala–scala communications^a^	–	SV-SM = 2.2 min
		ST-SV = 9,999 min
		ST-SM = 4.6 min
Scala–blood communications^a^	–	SV-blood = 1.6 min
		ST-blood = 1.6 min
		SM-blood = 15 min

Software settings and parameters that are not explicitly mentioned were applied in the standard configuration

^a^Software parameters defined on the basis of experimental data from [44])

**Table 2:** Results from baso-apical length measurements of AQP4 and AQP5 and AQP5 radial width immunofluorescence in OSCs in the half-turns VII and VIII of the adult guinea pig cochlea

	Baso-apical length of AQP4-IF (μm)	Baso-apical length of AQP5-IF (μm)	Radial width of AQP5-IF (μm)
	Half-turn VII		
Specimen 1	904.12	–	–
Specimen 2	953.16	–	–
Specimen 3	933.74	–	–
Mean value	930.34	–	–
SD	24.70	–	–
	Half-turn VIII		
Specimen 1	834.78	726.99	60.23
Specimen 2	819.30	872.73	57.93
Specimen 3	801.75	813.22	54.44
Mean value	818.61	804.31	57.53
SD	16.53	73.28	2.92

Measurements were taken on three specimens (1–3) derived from three independent animals

*IF* immunofluorescence, *SD* standard deviation

**Table 3:** Results of nonlinear regression analyses for the simulated time-dependent endolymphatic THO concentration change in the experimental models SV/SM, ST/SM and SV + ST/SM

	SV/SM	ST/SM	SV + ST/SM	In vivo SV + ST/SM (Konishi et al. [44])
*P*′ (min^−1^)	0.691	0.499	0.869	0.85
*P″* (min^−1^)	0.938	0.940	0.898	0.9
*α* (min^−1^)	0.504	0.435	0.549	0.4

The variables *P*′ (rate constant of THO exchange between the perilymph and endolymph), *P*″ (rate constant of endolymphatic water extrusion) and *α* (time constant) are given in Eq. (2)

For the simulation of THO dispersal in the SV + ST/SM model (Fig. 2a), a modified version of the Cochlear Fluids Simulator (V. 1.6i) was used. This model allowed simultaneous perfusion of both perilymphatic scalae according to the experimental setup from Konishi et al. [44]. To simulate diffusional THO exchange between one of the perilymphatic scalae (SV or ST) and the SM (models SV/SM and ST/SM; Fig. 2b, c), we defined an unphysiologically high value for the time constant “half-time of intercompartmental substance exchange between the SV and ST” in the Cochlear Fluids Simulator (“scala–scala communications”, Table 1) to avoid THO exchange between the SV and ST. The half-times of substance exchange between the SV and SM and between the ST and SM were adjusted by fitting the curve of endolymphatic THO concentration change to the endolymphatic THO concentration measured in vivo after 7 min of perilymphatic perfusion (Fig. 6a–c, data points *; Fig. 2a–c, SM in the diagrams). THO dispersal in the SV + ST/SM, SV/SM and ST/SM models was simulated for 10 min (Fig. 6a–c) and for 120 min (Fig. 6d–f) of perilymphatic perfusion to show the initial slope and the steady-state plateau of the endolymphatic THO concentration, respectively. The curves of the simulated endolymphatic THO concentration change from Fig. 6d–f were used for regression analyses to determine the rate constants of perilymphatic–endolymphatic THO (water) exchange (*P*′) for the three models (Fig. 6g–i).

## Results

Water permeability coefficients for the entire CDE, RM and OC were determined in this study based on previously derived in vivo experimental data on the diffusive [44] and osmotic water exchange [78] across these epithelial boundaries in the guinea pig cochlea. Konishi et al. [44] measured the diffusive uptake of radioactively labeled water (tritiated water, THO) into the endolymph during simultaneous (SV and ST) and separate perfusion of the perilymphatic scalae (SV or ST) with THO. During simultaneous THO perfusion of SV and ST (for further details see “Materials and methods” section; SV + ST/SM model, Fig. 2a), diffusive uptake of THO into the endolymph occurred across the entire CDE; during separate THO perfusion of SV (SV/SM model, Fig. 2b) or ST (ST/SM model, Fig. 2c), endolymphatic THO uptake occurred only across RM or OC, respectively. This data was used in the present study to determine *P*_D_ values for the entire CDE (SV + ST/SM model), RM (SV/SM model) and OC (ST/SM model).

Salt and DeMott [78] measured the concentration changes of the ionic volume marker (tetramethylammonium, TMA^+^) in the endolymphatic compartment during perilymphatic perfusion with media that were hyper- or hypoosmolar compared to the endolymph. During hyperosmolar perilymphatic perfusion, the osmotically driven outflow of water from the endolymphatic compartment was indirectly measured by the concentration increase of TMA^+^ (for further details, see “Materials and methods” section; Fig. 3a). This osmotically driven water outflow from the endolymphatic compartment induced two phenomena: (1) shrinkage of the entire endolymphatic compartment (Fig. 3b; presumably via transepithelial water outflow along the entire CDE) and (2) longitudinal endolymph flow towards the cochlear apex (Fig. 3c; via an unknown mechanism). These data were used in the present study to determine *P*_f_ values for the entire CDE (SV + ST/SM model) and a subpopulation of OSCs in the cochlear apex (OSC_apex_), respectively.

### Surface areas of the cochlear perilymph–endolymph barrier

**Fig. 4:**
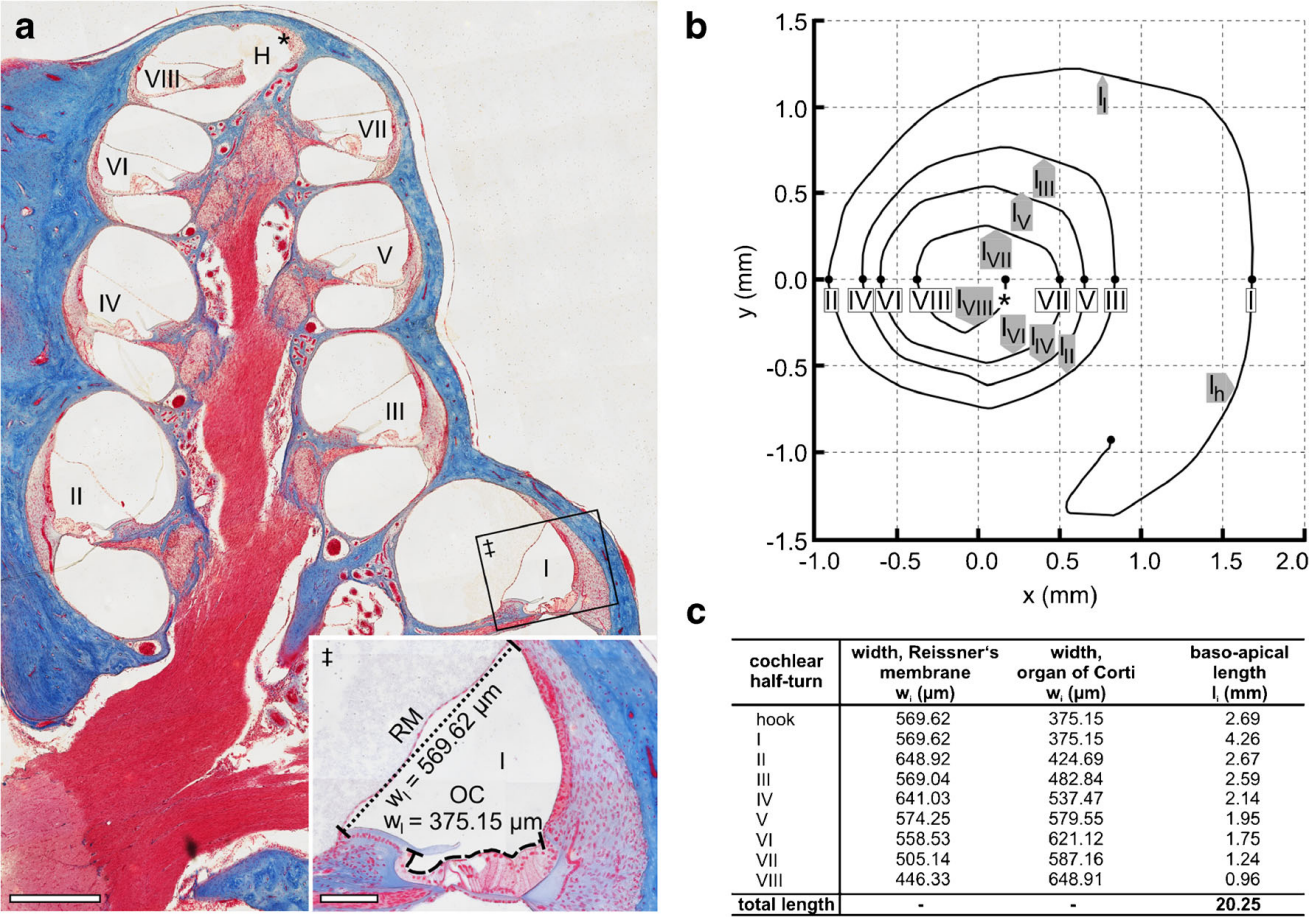
Determination of surface areas of the cochlear perilymph–endolymph barrier (PEB) on the adult guinea pig cochlea. **a** Overview of an azan-stained lateral (midmodiolar) section of the adult guinea pig cochlea. The width (radial length) of Reissner's membrane (*RM*) separating the SV from the SM (‡, *dotted line*) and the width of the apical surface of the organ of Corti (OC) separating the ST from the SM (‡, *dashed line*) were measured in all eight cochlear half-turns (W_I_–W_VIII_) (*H*, helicotrema; *asterisk*, apical end of the cochlear duct). **b** The baso-apical (longitudinal) length of RM and that of the OC were determined for each cochlear half-turn based on orthogonal plane fluorescence optical sectioning (OPFOS) data of the adult guinea pig cochlea, derived from Hofman et al. [32]. An OPFOS-based projection of the cochlear spiral in the XY plane (**b**, adapted from Hofman et al. [32] with permission from the corresponding author and the publisher, *Wiley*-*Blackwell*) was used to measure the longitudinal length of the eight cochlear half-turns (*l*_I_–*l*_VIII_) and the hook region (*l*_h_). **c** Results of width and length measurements determined in **a** and **b**. *Scale bars*: (**a**) 500 μm; (**a**, ‡) 100 μm

The water-permeated surface areas of the cochlear PEB in the models (1) SV + ST/SM, (2) SV/SM and (3) ST/SM were determined. The radial width of RM, separating SV and SM (black dotted line, Fig. 4a (inlay ‡); direct permeation barrier in the SV/SM model), and that of the OC, separating ST and SM (black dashed line, Fig. 4a (inlay ‡); direct permeation barrier in the ST/SM model), were measured in each of the eight cochlear half-turns (Fig. 4a, I–VIII). Additionally, we measured the length of the 8.5 cochlear half-turns (including the hook region) on a XY projection of the cochlear spiral that was derived from 3D reconstruction data of the guinea pig inner ear (Fig. 4b, adapted from [32]; reprinted with the kind permission from the corresponding author R. Hofman and the publisher *Wiley*-*Blackwell*). The results of the width and length measurements determined in Fig. 4a, b are shown in Fig. 4c.

The surface areas of RM and the OC were calculated using Eq. (1) for each of the 8.5 cochlear half-turns (*A*_I-VIII_) as follows:

1 $$ {A}_i={l}_i\times \frac{w_i+{w}_{i+1}}{2},\kern1em i=\kern0.5em h,I, II, II I, IV,V, VI, VI I, VI I I $$

where *h*, *I*, *II*, *III*, *IV*, *V*, *VI*, *VII* and *VIII* are the indices for the hook region (*h*) and the cochlear half-turns I–VIII, *l*_*i*_ is the baso-apical, longitudinal length of the *i*th cochlear half-turn (*l*_h_–*l*_VIII_, 2B and 2C) and *w*_*i*_ and *w*_*i* + 1_ are the widths of RM or the OC at the basal (*w*_*i*_) and apical end (*w*_*i* + 1_) of the corresponding half-turn (Fig. 4c). For the hook region, the width of RM and the width of the OC measured in the first cochlear half-turn (*w*_I_), multiplied by the length of the hook region (*l*_h_), was used. The calculated partial surface areas for RM (*A*_SV/SM, *i*_) and the OC (*A*_ST/SM, *i*_) from each cochlear half-turn and the hook region were summed to obtain the total surface sub-areas of *A*_SV/SM_ and *A*_ST/SM_ of the cochlear PEB (*A*_SV/SM_ + *A*_ST/SM_).

The results from these measurements were as follows:

*A*_SV/SM_ = 11.46 mm^2^ of the cochlear PEB separating the SV from SM (RM);

*A*_ST/SM_ = 9.78 mm^2^ of the cochlear PEB separates the ST from the SM (OC) and from this it follows that 21.24 mm^2^ separates the SV and ST from the SM (*A*_SV+ST/SM_).

### Membrane area of complementary membranous aquaporin expression in OSCs at the perilymph–endolymph barrier

**Fig. 5:**
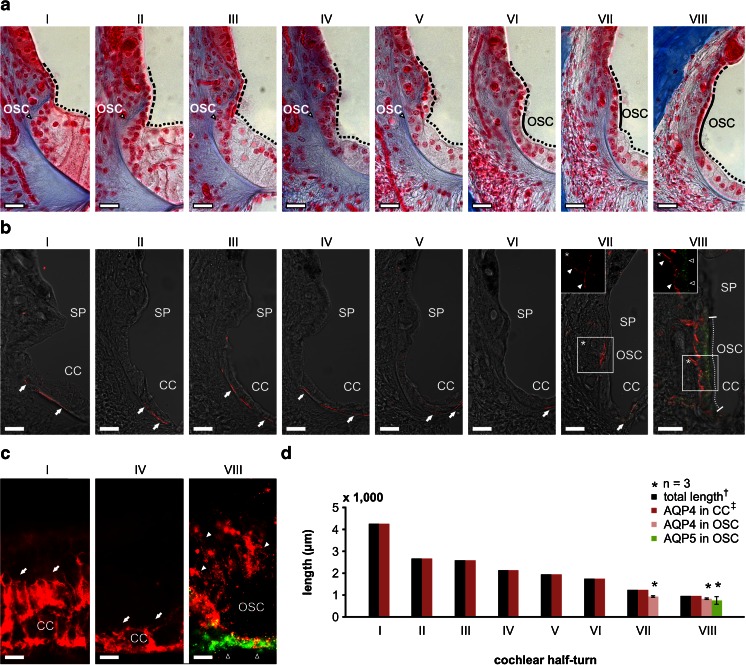
Complementary localisation of AQP4 and AQP5 in the apical and basolateral membranes of outer sulcus cells (*OSCs*) in the adult guinea pig cochlea. **a** The outer sulcus region in all eight cochlear half-turns (I–VIII) from azan-stained sections of the adult guinea pig cochlea. In cochlear half-turns I–V, the epithelial lining of the endolymphatic space in the outer sulcus region is formed by Claudius cells (CCs, *black dotted lines* mark the apical surface of CCs) and epithelial cells of the spiral prominence (SP, *black broken lines* mark the apical surface of SP epithelial cells). In these basal half-turns, OSCs are covered by CCs and the SP epithelial cells and therefore have no direct contact with endolymph. In contrast, in half-turns VI–VIII, OSCs are interposed between CCs and SP epithelial cells and thus are a direct constituent of the PEB. **b** Confocal images of immunofluorescence labelling of AQP4 (*red*) and AQP5 (*green*) in the outer sulcus region of the adult guinea pig cochlea. In half-turns I–VI, AQP4 labelling was detected in the basal membranes of CCs (*white arrows*). No immunoreactivity for AQP4 and AQP5 was detected in the OSCs of these half-turns. In half-turn VII, AQP4 labelling was observed in the basal membranes of CCs (*white arrow*), and OSCs showed AQP4 labelling in their basolateral membranes that enwrap their root processes (*, *white arrowheads*) but were devoid of AQP5 labelling. In the most apical half-turn (VIII), OSCs exhibited AQP4 labelling in their basolateral membranes (inlay *, *white arrowheads*) and AQP5 labelling in their apical membranes (inlay *, *hollow arrowheads*). The radial length of the apical membranes of OSCs in half-turn VIII that exhibited immunolabelling for AQP4 and AQP5 was measured as 56.19 ± 2.47 μm (VIII, *white dotted line*; *n* = 3). **c** Representative images of AQP4 (*red*) and AQP5 (*green*) immunolabelling on whole-mount preparations of the lateral wall in the half-turns I, IV and VIII that were used for baso-apical length measurements of AQP4 and AQP5 expression in OSCs. In the half-turns I and IV, AQP4 labelling was detected in CCs (*white arrows*). In the half-turn VIII, OSCs exhibited a polarised labelling of AQP4 (*white arrowheads*) and AQP5 (*hollow arrowheads*) in the basolateral root processes and at the apical side of the cells, respectively. **d** Quantification of the baso-apical length of AQP4 and AQP5 labelling in the outer sulcus region on whole-mount preparations of the cochlear lateral wall from each of the eight half-turns (I–VIII). AQP4 labelling in CCs was observed throughout the entire length of the whole-mount preparations. In OSCs, AQP4 labelling was restricted to a baso-apical length of 928.64 ± 34.68 μm (*n* = 3) in half-turn VII and 827.04 ± 10.95 μm (*n* = 3) in half-turn VIII. Additionally, in half-turn VIII, AQP5 labelling was detected in OSCs that also exhibited AQP4 labelling in their root processes along a baso-apical length of 749.86 ± 173.76 μm (*n* = 3). (*dagger*, the length of the entire cochlear lateral wall was derived from [32]; *double dagger*, the baso-apical length of AQP4 labelling in CCs was set equal to the total length of the lateral wall because we did not observe a baso-apical gradient of AQP4 labelling in CCs). *Scale bars*: (**a**, **b**) 20 μm, (**c**) 10 μm

Midmodiolar cryosections revealed that OSCs in cochlear half-turns I–V were covered by CCs at their apical pole and thus did not have direct contact with the endolymphatic space (Fig. 5a, I–V). In contrast, OSCs in the three most apical half-turns were interposed between CCs and spiral prominence (SP) epithelial cells; thus, the apical membranes of OSCs in half-turns VI–VIII had direct contact with the endolymph (Fig. 5a, VI–VIII). Immunolabelling of AQP4 and AQP5 on midmodiolar cryosections of the adult guinea pig cochlea revealed polarised membranous expression of AQP4 and AQP5 in OSCs in the cochlear apex (OSC_apex_). AQP4 labelling was present in the basolateral membranes (covering the root processes) of OSCs in cochlear half-turns VII and VIII (Fig. 5b, VII and VIII); AQP5 labelling was detected in the apical membranes and the subapical cytoplasm of OSCs and was restricted to the most apical half-turn (Fig. 5b, VIII).

The radial width of the apical membranes of OSCs that exhibited AQP5 expression as determined on immunolabelled cryosections was 57.53 ± 2.92 μm (Fig. 5b, VIII, white dotted line; Table 2; *n* = 3).

The longitudinal extent of complementary AQP4 and AQP5 expression in OSCs along the baso-apical length of the cochlear duct was determined from whole-mount preparations of the lateral wall from the adult guinea pig cochlea (Fig. 5c, d). Consistent with the results obtained from immunolabelled cryosections (Fig. 5b), AQP4 labelling in CCs was observed throughout the entire length of the cochlear duct (Fig. 5d, I–VIII). AQP4 labelling in OSCs was detected along the entire longitudinal length of the lateral wall of half-turn VIII (818.61 ± 16.53 μm, (Fig. 5d, VII; Table 2; *n* = 3) and further extended along 930.34 ± 24.70 μm in half-turn VII. Overlapping fluorescence signals for AQP4 and AQP5 were restricted to a distance of 804.31 ± 73.28 μm in the most apical half-turn (Fig. 5d, VIII; Table 2; *n* = 3).

The total length of the cochlear half-turns (Fig. 5d) was derived from length measurements of the adult guinea pig cochlear spiral (Fig. 4b, c [32]). The measurements on three whole-mount preparations of the lateral wall obtained from three different animals revealed a between-subject variation of the longitudinal extent of AQP4 and AQP5 labelling in OSCs of 2.02 and 9.10 % (measured values, see Table 2), respectively. These values are higher than the range of inter-individual variation determined for the length of the basilar membrane of the guinea pig cochlea described as 0.83 % [85]. This variance can be due to technical variations in the segmental whole-mount preparation of the lateral wall or a greater inter-individual variation in the longitudinal extend of AQP4 and AQP5 in the cochlear lateral wall.

The value of the radial width of the apical membranes of OSCs that exhibit complementary expression of AQP4 and AQP5 (57.53 ± 2.92 μm; Table 2; *n* = 3) was multiplied by the longitudinal membrane length of complementary expression of AQP4 and AQP5 in OSCs (804.31 ± 73.28 μm), yielding an OSC area (*A*_OSC_) of 46,271.95 μm^2^ (0.04627 mm^2^). This area represents a putative aquaporin-facilitated water shunt at the perilymph–endolymph barrier in the apex of the cochlea.

### Rate constants of diffusional water exchange at the cochlear perilymph–endolymph barrier

Konishi et al. [44] determined *P*′ for the entire cochlear PEB based on experimental in vivo data describing time-dependent endolymphatic uptake of THO during simultaneous THO perfusion of both perilymphatic scalae (SV + ST/SM model, Fig. 2a). In this model, Konishi et al. recorded the endolymphatic THO concentration in 1-min intervals, starting 3 min after the initiation of perilymphatic THO perfusion. The data points of the time-dependent endolymphatic THO concentration changes in the SV + ST/SM model were used by Konishi et al. for regression analysis based on Eq. (2):

2 $$ {C}_{\mathrm{e}}^{*}={C}_0^{*}\left[\frac{P^{\prime }}{P^{{\prime\prime} }}+\frac{\alpha {P}^{\prime }-0.5{P}^{\prime }{P}^{{\prime\prime} }}{P^{{\prime\prime}}\left({P}^{{\prime\prime} }-\alpha \right)}{e}^{-{P}^{\prime }t}-\frac{0.5{P}^{\prime }}{P^{{\prime\prime} }-\alpha }{e}^{-\alpha t}\right] $$

In Eq. (2), *C*_e_^*^ is the THO concentration in the endolymph, *C*_0_^*^ is the final concentration of THO in the perilymph, *P*′ is the rate constant of THO exchange between the perilymph and endolymph, *P*″ is the rate constant of THO outflow from the endolymphatic compartment and *α* is the time constant determining the slope of the change in the endolymphatic THO concentration. The least-squares fit to Eq. (2) by Konishi et al. [44] yielded *P*′ = 0.85 min^−1^ for the SV + ST/SM model. For the SV/SM and ST/SM models, no continuous measurements of endolymphatic THO concentration changes were performed by Konishi et al.; thus, *P*′ was not determined for the SV/SM and ST/SM models.

**Fig. 6:**
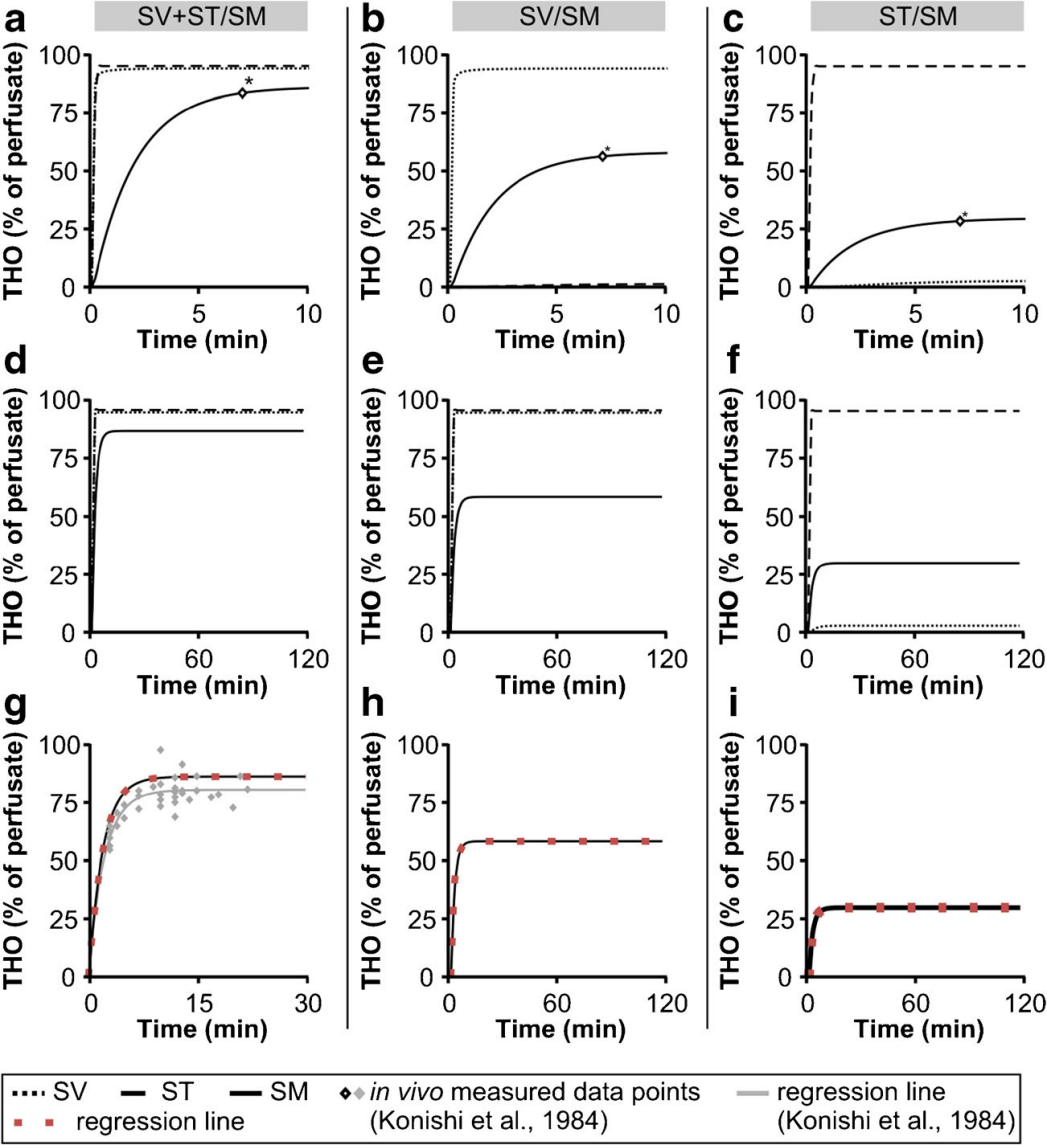
Simulations of time-dependent diffusional THO dispersal in the cochlear fluid compartments during perilymphatic tracer perfusion using the Cochlear Fluids Simulator (V. 1.6i). The program settings used for the simulations were adapted from the three experimental models SV + ST/SM, SV/SM and ST/SM ([44]; Fig. 2) and are provided in Table 1. **a**–**c** Simulations of the time-dependent THO dispersal in the perilymphatic and endolymphatic scalae during 10 min of perilymphatic THO perfusion in the SV and ST ((**a**), SV/SM), SV ((**b**), SV/SM) and ST ((**c**), ST/SM). Data points of endolymphatic THO concentration after 7 min (*asterisk*) were obtained from in vivo experiments [44] and were used to fit the curve of endolymphatic THO concentration change by adjusting the parameter “scala–scala communication” (Table 1). **d**–**f** Simulation settings from **a** to **c** were applied to simulate the steady-state conditions of THO dispersal during 120 min of perilymphatic perfusion in the models SV + ST/SM (**d**), SV/SM (**e**) and ST/SM (**f**). **g**–**i** Curves of endolymphatic THO concentrations from **d** to **i** (*black lines*) were used for regression analyses based on Eq. (2). The obtained regression curves that best fit the simulation data are shown as *red square lines*. In **g**, the in vivo measured data points of endolymphatic THO concentration (*grey squares*) and the corresponding regression line (*grey line*) derived from Konishi et al. [44] are shown. A comparison of the regression curve that was based on our in silico simulations (*red square line*) with the regression line derived from empirical data (*grey line*) revealed an almost identical slope and a similar plateau under steady-state conditions of both curves

In this study, we determined *P*′ for the entire cochlear PEB via in silico simulations with the Cochlear Fluids Simulator (V 1.6i, modified). The endolymphatic THO uptake derived from the SV + ST/SM model (Fig. 6g) was calculated as 0.869 min^−1^ (Table 3). This in silico value is consistent with the empirical in vivo value of 0.85 min^−1^ measured by Konishi et al. [44]. Hence, cochlear water dynamics simulations performed in silico also enabled calculations of *P*′ for the models in which perilymphatic scalae were separately perfused with THO. The data points of endolymphatic THO concentration measured in vivo 7 min after the onset of perilymphatic perfusion [44] were used in the in silico simulations for curve fitting. Based on the simulations performed in this study, *P*′ was calculated as 0.691 min^−1^ for the SV/SM model (Fig. 6h; Table 3) and 0.499 min^−1^ for the ST/SM model (Fig. 6i; Table 3). The in silico determined *P*′ values for the SV + ST/SM, SV/SM and ST/SM models were further used for *P*_D_ calculations.

### Diffusional water permeability coefficients of the cochlear perilymph–endolymph barrier

Calculations of *P*_D_ were based on surface quantifications of the cochlear PEB and in silico simulations of diffusional THO dispersal between the perilymphatic and endolymphatic scalae performed with the Cochlear Fluids Simulator (V 1.6i, modified). In our *P*_D_ calculations for the cochlear PEB, we made the following assumptions: (1) transepithelial THO permeation occurred only across RM in the SV/SM model, across the OC in the ST/SM model, and across both epithelial structures in the SV + ST/SM model; and (2) the continuous perilymphatic perfusion minimised unstirred layer effects, which therefore did not contribute significantly to the resistance of water diffusion across the plasma membranes of the cochlear duct epithelium; and (3) the pre-existing osmotic gradient between the endolymph (304.2 mOsm (kg H_2_O)^−1^ [44]) and perilymph (293.5 mOsm (kg H_2_O)^−1^ in the SV and 292.9 mOsm (kg H_2_O)^−1^ in the ST [44]) diminished with the onset of perilymphatic perfusion. Consistent with this assumption, Konishi et al. confirmed the absence of significant differences in osmolarity between the SM, SV and ST after perilymphatic perfusion by measuring the osmolarities in the cochlear scalae. Therefore, THO dispersal between the perilymphatic and endolymphatic spaces was driven only by the diffusional spread of the tracer and not by solvent drag.

**Fig. 7:**
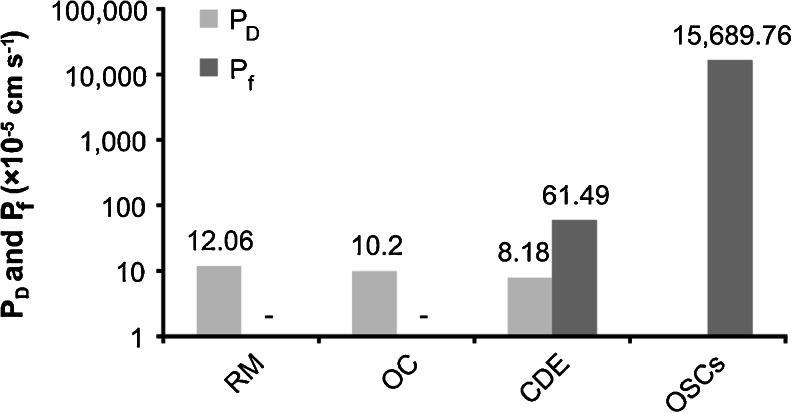
Results for calculated diffusional (*P*_D_) and osmotic water permeability coefficients (*P*_f_) of Reissner's membrane (*RM*; PEB between the SV and SM), the organ of Corti (*OC*; PEB between the ST and SM), the entire cochlear duct epithelium (*CDE*; entire cochlear PEB) and the apically located OSCs, which exhibited complementary membranous localisation of AQP4 in the basolateral membrane and AQP5 in the apical membrane. *y*-axis on the logarithmic scale

*P*_D_ values for the cochlear PEB were calculated using Eq. (3), which was derived from [44]:

3 $$ {P}_{\mathrm{D}}=\frac{P^{\prime }{V}_{\mathrm{e}}}{A} $$

where *P*′ is the rate constant of transepithelial diffusional water exchange between perilymph and endolymph, derived from our in silico simulations of the SV/SM, ST/SM and SV + ST/SM models. *V*_e_ is the endolymph volume (1.2 μl in the adult guinea pig cochlea [91]), and *A* is the water-permeated surface area of the PEB, which was determined in this study for the SV/SM (*A*_SV/SM_), ST/SM (*A*_ST/SM_) and SV + ST/SM (*A*_SV+ST/SM_) models. For RM, we found the following: *P*′ = 0.691 min^−1^ and *A*_SV/SM_ = 11.46 mm^2^. Substitution of these values into Eq. (3) yielded a value for *P*_D_ of 12.06 × 10^−5^ cm s^−1^ for RM (SV/SM model). For the OC (ST/SM model) with *P*′ = 0.499 min^−1^ and *A*_ST/SM_ = 9.78 mm^2^, we calculated *P*_D_ = 10.2 × 10^−5^ cm s^−1^. For the entire cochlear PEB (SV + ST/SM model) with *P*′ = 0.869 min^−1^ and *A*_SV+ST/SM_ = 21.24 mm^2^, we calculated *P*_D_ = 8.18 × 10^−5^ cm s^−1^ (Fig. 7).

### Osmotic water permeability coefficients of the cochlear perilymph–endolymph barrier

*P*_f_ calculations for the cochlear PEB were based on data on surface areas of the cochlear PEB, data on the membrane area of complementary AQP4/AQP5 expression in OSCs and in vivo experimental data on osmotically induced changes in endolymphatic volume (“Material and methods”, Fig. 2). For the *P*_f_ calculations performed in this study, the following assumptions were made: (1) the decrease in the area of the SM during hypertonic perfusion of SV and ST [78] was caused by an osmotically induced transepithelial volume flow from the endolymphatic to the perilymphatic space; (2) this transepithelial outflow of endolymph occurred throughout the entire baso-apical length of the cochlear duct, across RM and the OC; (3) the continuous perilymphatic perfusion minimised unstirred layer effects, which therefore did not contribute significantly to the resistance of water permeation across the plasma membranes of the cochlear duct epithelium; (4) the apically directed endolymph flow in the SM [78] was caused by an osmotically induced transepithelial bulk-volume flow from the endolymph to the perilymph across a water shunt consisting of AQP4/AQP5-expressing OSCs in the apex of the cochlear duct; (5) the experimentally determined increase in endolymphatic TMA^+^ induced by shrinkage of the endolymphatic compartment (area) and apically directed endolymph flow (movement) was considered to be approximately linear within 20 min of hypertonic perilymphatic perfusion (“Materials and methods”, Fig 10); and (6) after 20 min of hypertonic perilymphatic perfusion, the osmotic gradient between the perilymphatic and endolymphatic spaces remained constant. Consequently, an approximately linear increase in endolymphatic TMA^+^ resulted from the linear volume outflow.

The transepithelial volume flows (*J*_v_) per second (in cubic centimetre per second), which induced shrinkage of the endolymphatic compartment (area, *J*_v-area_) and apically directed endolymph flow (movement, *J*_v-movement_), were calculated using Eq. (4):

4 $$ {J}_{\mathrm{v}}=\left({V}_{\mathrm{e}}\cdot \frac{\mathrm{SI}}{100\%}\right)/20 $$

where *V*_e_ is the endolymph volume in the adult guinea pig cochlea (1.2 μl [91]) and SI is the relative solute (TMA^+^) increase induced by shrinkage of the endolymphatic compartment (SI_Area_ = 22.1 %) and longitudinal volume flow (SI_Movement_ = 12.29 %), as measured 20 min after the onset of hypertonic perilymphatic perfusion [78].

With *V*_e_ = 1.2 μl and SI_Area_ = 22.1 %, *J*_v-Area_ equals 13.26 × 10^−3^ μl min^−1^; with *V*_e_ = 1.2 μl and SI_Movement_ = 12.29 %, *J*_v-Movement_ is 7.37 × 10^−3^ μl min^−1^.

Calculations of *P*_f_ for the cochlear PEB were based on Eq. (5):

5 $$ {P}_{\mathrm{f}}=\frac{J_{\mathrm{v}}}{{\overline{V}}_{\mathrm{w}}\cdot A\cdot \varDelta c} $$

which is derived from [19], where *J*_v_ is the transepithelial net volume flow, ${\overline{V}}_{\mathrm{w}}$ is the partial molar volume of water (18 cm^3^ mol^−1^), *A* is the water-permeated surface area of the PEB and Δ*c* is the osmotic gradient between perilymph and endolymph. Because Salt and DeMott [78] perfused the perilymphatic compartments of the SV and ST with a solution that was made hypertonic (400 mOsm (kg H_2_O)^−1^) compared with the endolymph in the SM (306 mOsm (kg H_2_O)^−1^) by the addition of 94 mM sucrose (“Materials and Methods”, Fig. 3), Δ*c* between the endolymph and perilymph was 0.094 mol l^−1^. *P*_f_ for the entire cochlear PEB, based on the variables *A*_SV+ST/SM_ (21.24 mm^2^) and *J*_v-Area_ (13.26 × 10^−3^ μl min^−1^) and on Eq. (5), equals 6.15 × 10^−4^ cm s^−1^. The *P*_f_ for OSCs that exhibit complementary membranous expression of AQP4 and AQP5 (OSC_apex_) was determined based on the values of *A*_OSC_ (0.04627 mm^2^) and *J*_v-Movement_ (7.37 × 10^−3^ μl min^−1^) and on Eq. (5) and equals 156.90 × 10^−3^ cm s^−1^ (Fig. 7).

### Estimation of the membrane density of AQP5 water channels in the apical membranes of OSCs

The density of AQP5 water channel proteins in OSCs (*n*_AQP5_) was estimated based on the single channel water permeability for AQP5 (*P*_f-AQP5_ ~ 5 × 10^−14^ cm^3^ s^−1^ [100]) and the transepithelial *P*_f_ of OSC_apex_ (156.90 × 10^−3^ cm s^−1^) from this study. *n*_AQP5_ for OSC_apex_ was determined using Eq. (6) which was derived from Dobbs et al. [15]

6 $$ {n}_{\mathrm{AQP}5}=\frac{P_{\mathrm{f}-\mathrm{OSCs}}}{P_{\mathrm{f}-\mathrm{AQP}5}} $$

with 3.45 × 10^4^ μm^−2^.

## Discussion

This study assessed the diffusional (*P*_D_) and osmotic (*P*_f_) water permeability coefficients of the CDE in the guinea pig model. Based on these results, four lines of evidence (1–4) reveal the physiological relevance of AQP-mediated perilymphatic–endolymphatic water exchange in the mammalian cochlea:

1. Quantitative comparison of *P*_D_ and *P*_f_ for the entire cochlear duct epithelium yields water permeability coefficients in the same range seen in epithelia with aquaporin-facilitated water permeation
  A comparison of *P*_D_ (8.18 × 10^−5^ cm s^−1^) and *P*_f_ (6.15 × 10^−4^ cm s^−1^) for the entire CDE with water permeability coefficients reported for various other AQP-expressing epithelia revealed that these values were of the same order of magnitude while non-AQP-expressing cells demonstrate much lower values (Fig. 8). The *P*_D_ and *P*_f_ values that most closely resemble those of the CDE are those reported for the conjunctival epithelium of the ocular bulb (*P*_D_ = 13 × 10^−5^ cm s^−1^ [11]; *P*_f_ = 11 × 10^−4^ cm s^−1^ [55]; Fig. 8). Notably, both the CDE and the conjunctival epithelium are classified as “tight” barriers [10, 33, 36, 44]. Water flow across these tight epithelia thereby occurs via a transmembranous route and is most likely facilitated by AQPs that are expressed in the epithelial membrane domains (apical and basolateral) that face the two separated extracellular fluid compartments at the ocular surface (tear film and extracellular fluid in the conjunctival stroma) and in the cochlea (endolymph and perilymph), respectively. In the conjunctival epithelium (and in the corneal epithelium that is continuous with the conjunctival epithelium), AQP3 [27, 55, 71] in the baso-lateral domain [39] and AQP5 [67] at the apical ocular surface domain [39] determine the primary route of transepithelial water permeation as indicated by a four- and fivefold reduction in membrane osmotic water permeability (*P*_f_) in transgenic mice lacking AQP3 or AQP5 water channels, respectively [55].

**Fig. 8:**
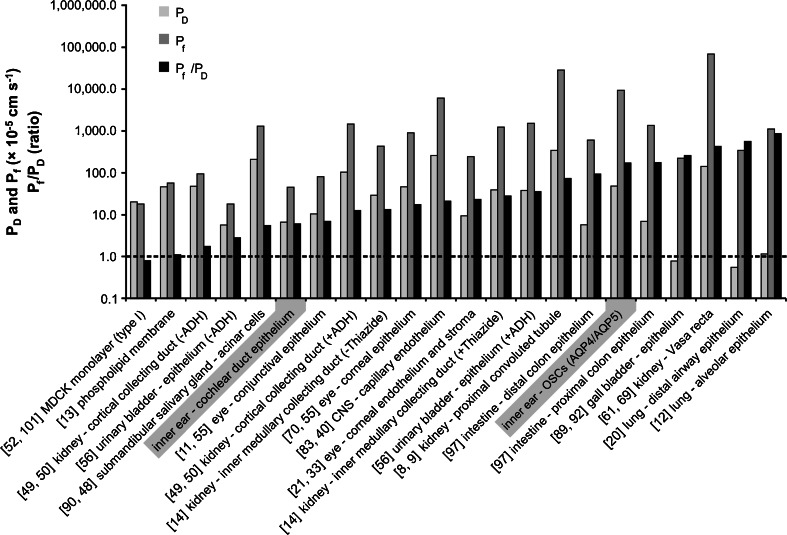
**a** Comparison of transepithelial diffusional (*P*_D_) and osmotic water permeability (*P*_f_) coefficients of AQP-expressing endo-/epithelia, including the values of the entire cochlear duct epithelium and the AQP4/5-expressing outer sulcus cells in the cochlear apex that were determined in this study. The *P*_f_/*P*_D_ ratios were calculated in this study. *y*-axis on the logarithmic scale; references are given in the figure
  In the CDE, a complementary membraneous localisation of AQPs (AQP4 and AQP5) has only been established for the OSCs in the outer sulcus region of the cochlear apex [18, 30, 31, present study]. However, for many other cell types in the CDE, the expression of multiple AQP subtypes has been described (reviewed in [17]), e.g. AQP7 and AQP9 in the epithelium of RM that separates the endolymph in the SM and perilymph in the SV, and AQP2, AQP4, AQP5, AQP7 and AQP9 in supporting cells of the OC that divides the endolymph in the SM and perilymph in the ST. Although a complementary localisation of AQPs in the basolateral and apical membranes of these cells has not been established, it appears likely that further analysis will obtain additional complementary expression patterns at the subcellular level. The presence of multiple AQPs in most of the cells types in the tight CDE and the similarity of *P*_D_ and *P*_f_ values suggest transmembranous AQP-facilitated water permeation across the entire CDE, as has been experimentally demonstrated for multiple AQP-expressing epithelia (Fig. 8).
  In summary, the presence of several AQP subtypes in various cells of the CDE and the similarity between *P*_D_ and *P*_f_ values of the entire CDE and multiple other AQP-expressing epithelia strongly suggest a transmembranous AQP-facilitated water permeation across the entire CDE.
2. The ratios of *P*_f_/*P*_D_ determined for the cochlear duct epithelium and aquaporin-expressing outer sulcus cells account for an aqueous pore-mediated water permeation
  The ratio of osmotic-to-diffusional water permeability (*P*_f_/*P*_D_) exceeds unity (*P*_f_/*P*_D_ >1) in the case of an aqueous pore (e.g. AQP)-facilitated water permeation [19]. In this study, the ratio *P*_f_/*P*_D_ for the entire CDE was determined to be 7.52 (Fig. 8), a value that clearly exceeds 1. In contrast, epithelia that do not exhibit AQP-facilitated water permeation, such as MDCK type I monolayers ([52]; Fig. 8) or pure phospholipid-bilayer membranes ([13]; Fig. 8), exhibit a *P*_f_/*P*_D_ ratio that equals unity (*P*_f_/*P*_D_ ~1). At these interfaces, water permeation occurs by solubility–diffusion through the lipid bilayers rather than through aqueous pores [19]. All other epithelia listed in Fig. 6 express multiple AQPs in a complementary membranous distribution. Consistent with AQP-facilitated transepithelial water permeation, these epithelia exhibit *P*_f_/*P*_D_ ratios >1, with values ranging from 2.03 for the (non-ADH-stimulated) kidney cortical collecting duct ([49]; Fig. 8) to 1,307.69 for the lung alveolar epithelium ([20]; Fig. 8). The *P*_f_/*P*_D_ ratio, determined for the entire CDE (7.52), closely resembles the value of the conjunctival epithelium (*P*_f_/*P*_D_ = 8.46; Fig. 8) or the submandibular salivary gland epithelium (*P*_f_/*P*_D_ = 6.67; Fig. 8). Notably, in both of these epithelia, AQP5 water channels are localised in the apical cell membranes [24, 55, 60] and determine the rate-limiting barrier for transepithelial water permeation (in case of the salivary gland acinar cells under pilocarpine-stimulated saliva secretion [48, 55, 59]).
  To determine the ratio *P*_f_/*P*_D_ for the subdomain of AQP5-expressing OSCs in the cochlear apex (*P*_f_/*P*_D_ = 242.02, Fig. 8), no in vivo experimental data was available to determine its *P*_D_; moreover, the Cochlear Fluids Simulator (V 1.6i, modified) did not allow for diffusive water dynamics simulations in a delimited area of the cochlear duct, such as the cochlear apex where AQP5-expressing OSCs are located. We therefore assumed a *P*_D_ for these cells (*P*_D-OSCs_ = 64.83 × 10^−5^ cm s^−1^), which represents a mean of the experimentally determined *P*_D_ values of AQP5-expressing epithelia, i.e. the lung alveolar epithelium (1.30 × 10^−5^ cm s^−1^ [12]), the corneal epithelium (1.68 × 10^−5^ cm s^−1^ [21]), the cochlear duct epithelium (8.18 × 10^−5^ cm s^−1^, present study), the conjunctival epithelium (13 × 10^−5^ cm s^−1^ [11]) and the salivary gland acinar epithelium (300 × 10^−5^ cm s^−1^ [90]). We did not apply the in silico determined *P*_D_ of the entire CDE for AQP5-expressing OSCs, since all other cell types in the CDE do not exhibit AQP expression in their apical membranes. Using the *P*_D_ of the entire CDE for AQP5-expressing OSCs would therefore most likely result in an underestimation of its *P*_D_, and an overestimation of the *P*_f_/*P*_D_ ratio. The *P*_D_ values from different AQP5-expressing epithelia differ from each other by 2 orders of magnitude, and the *P*_D_ of OSCs presumably is within this range. However, due to missing data on the diffusive water permeability of OSCs and the high variability of the *P*_D_ values from other AQP5-expressing epithelia, we can only achieve a rough estimate of the *P*_D_ of AQP5-expressing OSCs.
  The *P*_f_/*P*_D_ ratio estimated for AQP5-expressing OSCs closely resembles the value determined for the renal proximal tubule epithelium, as well as the proximal and distal colon epithelium (Fig. 8) in which the transepithelial fluid reabsorption largely depends on AQP-facilitated water permeation.
  In summary, the ratios *P*_f_/*P*_D_ determined for the entire CDE (*P*_f_/*P*_D_ = 7.52) and the AQP4/AQP5-expressing subpopulation of OSCs in the cochlear apex (*P*_f_/*P*_D_ = 242.02) clearly exceed 1, and therefore indicate aqueous pore-facilitated transepithelial water permeation between the perilymph and endolymph. A comparison of both values reveals that the different cell types of the CDE apparently do not uniformly contribute to transepithelial water permeation. The exceedingly high ratio determined for AQP-expressing OSCs indicates a high-transfer region for transepithelial water permeation in the cochlear apex.
3. The absolute *P*_f_ values determined for the entire CDE and the epithelial subdomain of OSCs in the cochlear apex reveal a highly permeable AQP4/AQP5-based water shunt in the cochlear apex
  An absolute value of *P*_f_ greater than 1 × 10^−2^ cm s^−1^ indicates aqueous pore-facilitated water permeation [94]. For the entire CDE, we determined a considerably lower *P*_f_ of 6.15 × 10^−4^ cm s^−1^; however, the cochlear duct is a highly heterogeneous epithelium that consists of 12 morphologically and functionally diverse cell types. Many of these cell types exhibit distinct AQP expression patterns, while others are devoid of AQP expression (reviewed in [17]). Hence, the cellular constituents of the CDE apparently exhibit distinct water permeability characteristics and the *P*_D_ and *P*_f_ values calculated in this study for the entire CDE, RM and the OC therefore represent average values of the diffusional and osmotic water permeabilities in the CDE.
  In this study, we determined the *P*_f_ of a single cell type of the CDE, a subpopulation of AQP4/AQP5-expressing OSCs in the cochlear apex, to be 156.90 × 10^−3^ cm s^−1^ (Fig. 7). This exceptionally high *P*_f_ value is 280-fold higher than the average *P*_f_ of the entire CDE (6.15 × 10^−4^ cm s^−1^; Fig. 7) and clearly exceeds the value of 1 × 10^−2^ cm s^−1^, which indicates aqueous pore-facilitated water permeation across OSCs. In accordance with the results from previous light microscopic [84] and ultrastructural [16] studies, we found this subpopulation of OSCs in the most apical half-turns (VI–VIII) of the guinea pig cochlea to be exclusively interposed between CCs and SP epithelial cells, thereby representing direct constituents of the cochlear PEB. Moreover, in this study, the complementary membranous localisation of AQP4 (basolateral) and AQP5 (apical) was demonstrated in this particular subpopulation of OSCs of the cochlear apex. The complementary subcellular distribution of AQP4 and AQP5 in this specific subpopulation of apically located OSCs has previously been described in the cochleae of other mammalian species, in particular rat [31] and human [18, 31]. As AQPs in the membranes of this subpopulation of OSCs putatively mediate transmembranous water exchange between the endolymph and the OSC cytoplasm (AQP5), as well as between the OSC cytoplasm and the perilymphatic extracellular fluid of the spiral ligament (AQP4), they form the molecular basis for an AQP-facilitated “water shunt” between the endolymph and perilymph in different mammalian species, as previously proposed [17, 31].
  Quantitatively, the *P*_f_ of OSCs is comparable to the *P*_f_ of the kidney proximal convoluted tubule (PCT) epithelium (500 × 10^−3^ cm s^−1^, Fig. 8 [9]). In the PCT epithelium, AQP1 in the apical and basolateral membranes [64], and AQP7 in the apical membranes [34] represent the major constituents of membrane osmotic water permeability [81, 86] and thereby facilitate the reabsorption of 50–60 % of the fluid filtered by the glomeruli (i.e. ~98 l per day), in order to maintain whole-body water homeostasis. The *P*_f_ value of OSCs in the cochlear apex, which matches that of the PCT epithelium, suggests a high water transport capacity for OSCs that are potentially relevant for cochlear water homeostasis.
  In summary, the *P*_f_ values determined for the entire CDE and the epithelial subdomain of AQP4/AQP5-expressing OSCs in the cochlear apex differ by 2 orders of magnitude. This suggests highly heterogeneous water permeability characteristics among the different cell types in the CDE. For OSCs in the cochlear apex, which are a direct constituent of the PEB, we propose a high capacity AQP water shunt that enables passive bulk water movements between the endolymph and perilymph contributing to longitudinal flow.
4. The estimated AQP5 channel density in the apical membranes of OSCs substantiates a high-transfer water shunt in the cochlear apex
  The density of AQP5 channel proteins in the apical membranes of OSCs in the cochlear apex was estimated to be 3.45 × 10^4^ μm^−2^. This value represents the characteristic AQP membrane density of 10^3^–10^4^ μm^−2^ as found in many cell membranes [93], and is in particular close to the membrane density of AQP5 channels determined in the alveolar epithelium (1.4 × 10^4^ μm^−2^ [15]). Notably, these characteristic AQP membrane densities exceed that of ion channels in various cell membranes generally quantified as 10^1^–10^2^ μm^−2^ [29] by several orders of magnitude.
  Water flow across the water shunt in OSCs of the cochlear apex is presumably rate-limited by AQP5 in the apical membrane, since (1) the basolateral membrane that enwraps the root processes of OSCs forms a larger membrane area for water permeation, and (2) the single channel water permeability of AQP4 (*P*_f-AQP4_ ~25 × 10^−14^ cm^3^ s^−1^ [100]) in the basolateral membranes of OSCs is five times higher than that of AQP5 (*P*_f-AQP5_ ~5 × 10^−14^ cm^3^ s^−1^ [100]).
  In summary, the estimated membranous AQP5 channel density in OSCs provides a plausible molecular basis for the high osmotic water permeability determined for this epithelial subdomain in the CDE.

### Putative physiological significance of the aquaporin-facilitated water shunt in outer sulcus cells in longitudinal endolymph homeostasis

A putative driving force for transcellular water movement across the AQP water shunt in OSCs of the cochlear apex is the osmotic gradient of 11 mOsm (kg H_2_O)^−1^ between the perilymph (293 mOsm (kg H_2_O)^−1^ [44]) and endolymph (304 mOsm (kg H_2_O)^−1^ [44]), as measured in the guinea pig cochlea under physiological conditions. This osmotic gradient is primarily determined by the ionic composition of the inner ear fluids and by the permeability of the cochlear PEB to ions [10, 43–45]. Alterations in the ion content in inner ear fluids, e.g. by the systemic administration of glycerol [38] or by disturbance of the ionic permeability of the PEB (e.g. induced broadband noise [46], low-frequency sound stimulation [76] or hypothermia [47]), affect the perilymphatic–endolymphatic osmotic gradient and thus affect the driving force for transepithelial water movement between the fluid compartments. At the molecular level, it was shown that acoustic stimulation leads to an increase in the endolymphatic ATP concentration, which presumably stimulates a purinergic (P2X_2_) receptor-mediated K^+^ outflow from endolymph via ion channels in the apical and basolateral membranes of OSCs [54]. The OSCs thereby constitute a regulated (“parasensory”) transepithelial K^+^ pathway that is thought to regulate K^+^ efflux through sensory hair cells during changes in the level of acoustic stimulation [54] and protect from exposure to noise [99]. Accompanying passive water fluxes across OSCs via AQP4 and AQP5 in their basolateral and apical membranes potentially equilibrate local osmotic shifts that arise from P2X_2_ receptor-regulated transcellular K^+^ fluxes across OSCs.

**Fig. 9:**
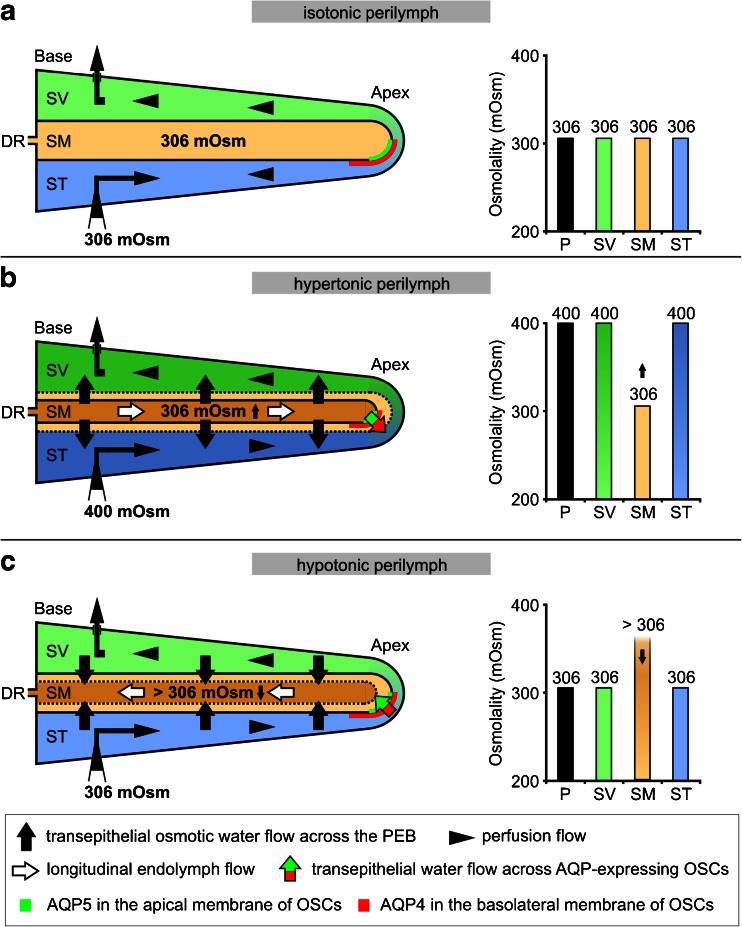
Hypothetical mechanisms of osmotically induced, AQP4- and AQP5-dependent transepithelial water flow in the cochlear apex and the effect of this flow on longitudinal endolymph flow. **a** In the in vivo experiments by Salt and DeMott [78], perfusion of the scala tympani (*ST*) and the scala vestibuli (*SV*) with a solution that was iso-osmotic to the endolymph (306 mOsm (kg H_2_O)^−1^) had no effect on endolymph volume or longitudinal endolymph flow in the scala media (*SM*). **b** Hyperosmotic (400 mOsm (kg H_2_O)^−1^) perilymphatic perfusion induced shrinkage of the endolymphatic compartment, presumably via osmotically induced transepithelial water outflow along the entire cochlear duct epithelium. Moreover, the longitudinal endolymph flow was into the blind-ending apex of the cochlear duct. This longitudinal flow can be explained by a bulk outflow of water from endolymph across the PEB in the cochlear apex, facilitated by AQP5 in the apical membranes and AQP4 in the basolateral membranes of OSCs in direct contact with the endolymph. **c** When the perilymphatic perfusion solution was changed back to 306 mOsm (kg H_2_O)^−1^, a partial recovery of endolymph volume was measured by Salt and DeMott [78]. This endolymphatic volume increase was potentially induced by transepithelial water inflow along the entire cochlear duct epithelium in response to a reversed perilymphatic–endolymphatic gradient (volume outflow in **c** led to an increase in the levels of endolymphatic osmolytes (>306 mOsm (kg H_2_O)^−1^)). Consequently, the observed basally directed longitudinal endolymph flow during rehydration of the endolymphatic space [78] may be based on the osmotically induced transepithelial bulk inflow of endolymph through AQP4 and AQP5 in the membranes of OSCs in the apex of the cochlear duct epithelium (*DR*, ductus reuniens)

Experimental studies in vivo have shown that the volume of cochlear endolymph was reduced by perfusing the perilymphatic spaces with hypertonic medium and at the same time induced an apically directed endolymph flow of 2.5 nl min^−1^ ([78]; Fig. 9b). Similarly, when the osmotic gradient between the perilymph and endolymph was reversed, a slower basally directed endolymph flow was observed ([78]; Fig. 9c). This basally directed endolymph flow was also induced by acute volume injections into the cochlear endolymph [77]. Notably, even under the physiological conditions with an osmotic gradient between perilymph and endolymph (11 mOsm (kg H_2_O)^−1^ [44]), the mean rate of endolymph flow directed toward the base was calculated to be 0.36 nl min^−1^; this value has been classified as not significantly different from zero and therefore indicates the absence of longitudinal endolymph flow under normal conditions [80]. These in vivo experimental findings were in line with the long-standing “dynamic flow theory” for endolymph, which was introduced by Lawrence et al. [53]. This theory attempts to explain the fundamental mechanisms of endolymph generation and homeostasis by combining the concepts of local “radial flow” of endolymph [62] as the predominant homeostatic mechanism under physiological conditions (indicated by the absence of longitudinal flow [80]) and the “longitudinal flow” [25] as a compensatory homeostatic mechanism that occurs exclusively in disturbed endolymphatic fluid (indicated by longitudinal flow when endolymph volume [77] or perilymphatic–endolymphatic gradients are altered [78]); however, experimental evidence for these proposed mechanisms of endolymph homeostasis is sparse. The longitudinal endolymph flow in vivo was extremely low in the normal animal [80] but could be induced by experimental manipulations [77, 78]. Conversely, the radial endolymph flow has never been demonstrated experimentally. Large radial currents have been demonstrated [102] and it is widely accepted that major ions, such as K^+^, are recycled [88]; however, volume flows associated with local ionic circulation are thought to be minimal and hence endolymph is not secreted in volume. Moreover, no molecular determinants in the cells of the cochlear duct epithelium have been described that account for endolymph volume movements.

**Fig. 10:**
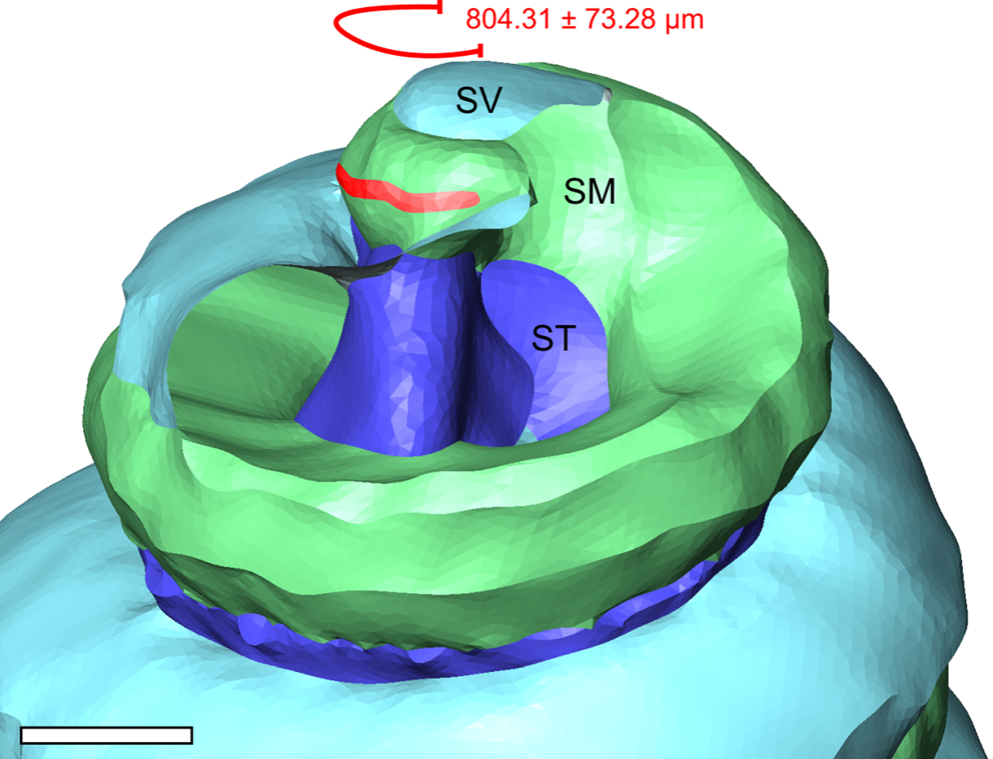
Anatomical localisation of the AQP water shunt in OSCs, illustrated in an orthogonal-plane fluorescence optical sectioning (OPFOS)-based three-dimensional reconstruction of the helicotrema region in the adult guinea pig cochlea. The AQP water shunt (*red outline*) communicates along a distance of 804.31 ± 73.28 μm with the endolymph in the scala media (*SM*; *green outline*) and with the perilymph at the confluence of the scala vestibuli (*SV*; *light blue outline*) and the scala tympani (*ST*; *dark blue outline*) in the helicotrema. *Scale bar*, 500 μm

Here, we propose that bidirectional longitudinal endolymph movements generated under conditions of an endolymph volume disturbance (as demonstrated experimentally in vivo [78]) are induced by osmotic bulk water flow out of (apically directed endolymph flow, Fig. 9b) and into (basally directed endolymph flow, Fig. 9c) the endolymphatic fluid compartment through the AQP-facilitated water shunt in the cochlear apex at the helicotrema (Fig. 10). The AQP-facilitated water shunt thereby enables OSCs in the cochlear apex to function to secrete and resorb endolymph as postulated in previous studies using this cell type [2, 84, reviewed in 35]; these results support the contribution of longitudinal flow to endolymph homeostasis in the cochlea.

### Putative pathophysiological significance of the aquaporin-facilitated water shunt in outer sulcus cells in disturbed inner ear fluid homeostasis (e.g. Ménière's disease)

The disturbance of longitudinal endolymph flow is regarded as an essential component of the formation of endolymphatic hydrops (EH). As idiopathic EH is an obligatory histopathological finding in Ménière's disease [26, 98], the disturbance of longitudinal endolymph flow has been considered a critical component of the aetiology of this inner ear affliction. In attempts to identify the underlying cause of Ménière's disease, the results of numerous morphological and functional studies pointed to the endolymphatic sac (ES) as the main site causing the disturbance of endolymphatic-volume homeostasis [25, 41, 57, 63]; however, a failure in active secretion and/or resorption of the endolymph by the ES fails to conclusively explain morphological and functional alterations localised in the cochlear apex of Ménière's disease animal models and temporal bone specimens derived from Ménière's disease patients. For example in post-mortem human temporal bone specimens, EH in the apical turn of the cochlea is the first histopathomorphologic sign and was observed in each of the 95 cases investigated in a study by Pender [72]. Later during disease progression, EH becomes most severe in the cochlear apex and further extends to other portions of the membranous labyrinth [68, 72]. Furthermore, degenerative changes in sensory and neural elements, as well as functional impairment (i.e. low-frequency hearing loss), primarily manifest in the cochlear apex. These pathological abnormalities were attributed to chronic alterations in the local fluid environment of the cochlear apex [82]. Ménière's disease-specific abnormalities in the cochlear apex that explain these morphological and functional impairments have not yet been described at the molecular level.

Recently, molecular–epidemiologic studies investigating the association of AQP5 gene polymorphisms with susceptibility to Ménière's disease [5, 66] identified a single-nucleotide polymorphism (SNP) in the AQP5 gene (rs3736309) that was associated with a reduced risk of Ménière's disease [66]. In other studies, carriers of the same SNP were shown to have a reduced risk of chronic obstructive pulmonary disease (COPD) [28, 65]. Serious symptoms of COPD are mucus hypersecretion and lung oedema, indicating the pathophysiological significance of airway submucosal gland epithelia (secreting most of the airway mucus) and the distal lung epithelium (reabsorbing the alveolar fluid) in COPD. In both airway epithelia, AQP5 expressed in the apical membranes determines the rate-limiting barrier for transepithelial fluid transport [58, 87]. These findings suggest a protective effect of the AQP5 SNP against lung fluid imbalances caused by AQP5-dependent transepithelial water permeation in COPD. Analogously, in the cochlea, the AQP5 SNP most likely alters the water permeability of the AQP4/AQP5 water shunt in OSCs of the cochlear apex and may therefore prevent EH formation and hence reduce the risk for Ménière's disease. These epidemiologic studies, in conjunction with the molecular and in silico functional characterisation of the AQP4/AQP5 water shunt in OSCs in this study, imply that it has a role in the pathophysiology of EH formation and Ménière's disease. Furthermore, pharmacological interventions aimed at the AQP4/AQP5 water shunt in the cochlear apex may form a basis for future medical therapies in Ménière's disease.

In conclusion, we showed that in the mammalian cochlea the diffusive (*P*_D_) and osmotic (*P*_f_) water permeability coefficients of the entire cochlear duct epithelium indicate aqueous pore-mediated transepithelial water permeation between the endolymph and perilymph. We further identified a high-transfer region for osmotic water flow between the endolymph and perilymph in the cochlear apex, constituted by an epithelial subdomain of outer sulcus cells that express the water channel proteins AQP4 and AQP5 in their basolateral and apical membranes, respectively. Bulk water flow across this AQP water shunt putatively drives osmotically induced longitudinal endolymph flows and may be of pathophysiologic significance in the generation of endolymphatic hydrops in Ménière's disease.

## Abbreviations

*A*_OSC_: Membrane area of OSCs that co-express AQP4 in their apical membranes and AQP5 in their basolateral membranes (in square micrometre)
*A*_ST/SM_: Water-permeated surface area of supporting cells of the organ of Corti (in square micrometre)
*A*_SV/SM_: Water-permeated surface area of Reissner's membrane (in square micrometre)
*A*_SV/SM_ + *A*_ST/SM_: Water-permeated surface area of the entire cochlear duct (in square micrometre)
ADH: Anti-diuretic hormone
AQP: Aquaporin
*α*: Time constant
CCs: Claudius cells
CDE: Cochlear duct epithelium
Cl^−^: Chloride
COPD: Chronic obstructive pulmonary disease
*C*^*^_0_: Final concentration of THO (tritiated water) in the perilymph (in mole per litre)
*C*^*^_e_: THO (tritiated water) concentration in endolymph
Δ*c*: Osmotic gradient between perilymph and endolymph (in mole per litre)
DAPI: 4′,6-diamidino-2-phenylindole
EDTA: Ethylenediaminetetraacetic acid
EH: Endolymphatic hydrops
ES: Endolymphatic sac
H_2_O: Water
*J*_v-Area_: Transepithelial volume outflow from endolymph that induced shrinkage of the endolymphatic compartment (in cubic centimetre per second)
*J*_v-Movement_: Transepithelial volume outflow from endolymph that induced longitudinal endolymph flow (in cubic centimetre per second)
K^+^: Potassium
*l*_*i*_: Baso-apical (longitudinal) length of the cochlear half-turn “*i*”
MDCK (type I): Madin–Darby canine kidney (type I) epithelium
mOsm: Milliosmole
*n*_AQP5_: Cell membrane density of AQP5 water channel proteins
Na^+^: Sodium
NDS: Normal donkey serum
OC: Organ of Corti
OSCs: Outer sulcus cells
*P*_D_: Diffusional water permeability coefficient (in centimetre per second)
*P*_f_: Osmotic water permeability coefficient (in centimetre per second)
*P*′: Rate constant of perilymphatic–endolymphatic THO exchange (in per minute, according to [44])
*P*″: Rate constant of endolymphatic water extrusion (in per minute, according to [44])
PBS: Phosphate-buffered saline
PCT Epithelium: Proximal convoluted tubule epithelium
PEB: Perilymph–endolymph barrier
PFA: Paraformaldehyde
RM: Reissner's membrane
SI_Area_: Endolymphatic solute (TMA^+^) increase induced by shrinkage of the endolymphatic compartment (according to [78])
SI_Movement_: Endolymphatic solute (TMA^+^) increase induced by longitudinal endolymph flow (according to [78])
SL: Spiral ligament
SM: Scala media
SNP: Single-nucleotide polymorphism
SP: Spiral prominence
ST: Scala tympani
ST/SM: Experimental model with THO perfusion of ST to determine *P*′ for OC
SV: Scala vestibuli
SV/SM: Experimental model with THO perfusion of SV to determine P´ for RM
SV + ST/SM: Experimental model with THO perfusion of SV and ST to determine *P*′ for the entire CDE
*t*: Time (in minute)
THO: Tritiated water (^3^H_2_O)
TMA^+^: Tetramethylammonium
*V*_e_: Endolymph volume (in microlitre)
*V*_w_: Partial molar volume of water (18 cm^3^ mol^−1^)
*w*_*i*_: Width of RM or the OC at the basal end of the cochlear half-turn “*i*”
*w*_*i* + 1_: Width of RM or the OC at the apical end of the cochlear half-turn “*i*”

## References

[1] Adamzik M, Frey UH, Bitzer K, Jakob H, Baba HA, Schmieder RE, Schneider MP, Heusch G, Peters J, Siffert W (2008). A novel-1364A/C aquaporin 5 gene promoter polymorphism influences the responses to salt loading of the rennin–angiotensin–aldosterone system and of blood pressure in young healthy men. Basic Res Cardiol.

[2] Altmann F, Waltner JG (1947). The circulation of the labyrinthine fluids; experimental investigations in rabbits. Ann Otol, Rhinol, Laryngol.

[3] Andersen HC (1948). Passage of trypan blue into the endolymphatic system of the labyrinth. Acta Otolaryngol.

[4] Angelborg C (1974). Distribution of macromolecular tracer particles (Thorotrast-r) in the cochlea. An electron microscopic study in guinea pig. Part I. The organ of Corti, the basilar membrane and the tympanic covering layer. Acta Otolaryngol Suppl.

[5] Arweiler-Harbeck D, Saidi F, Lang S, Peters J, Siffert W, Adamzik M (2012) The -1364A/C aquaporin 5 gene promoter polymorphism is not associated with Menière's disease. ISRN Otolaryngology. doi:10.5402/2012/706896 10.5402/2012/706896 PMC3671710 23762616

[6] Benga G (2009). Water channel proteins (later called aquaporins) and relatives: past, present, and future. IUBMB Life.

[7] Benga G, Borza T (1995). Diffusional water permeability of mammalian red blood cells. Com Biochem Physiol B Biochem Mol Biol.

[8] Berry CA (1985). Characteristics of water diffusion in the rabbit proximal convoluted tubule. Am J Physiol.

[9] Berry CA, Verkman AS (1988). Osmotic gradient dependence of osmotic water permeability in rabbit proximal convoluted tubule. J Membr Biol.

[10] Bosher SK, Warren RL (1968). Observations on the electrochemistry of the cochlear endolymph of the rat: a quantitative study of its electrical potential and ionic composition as determined by means of flame spectrophotometry. Proc R Soc Lond B Biol Sci.

[11] Candia OA, Shi XP, Alvarez LJ (1998). Reduction in water permeability of the rabbit conjunctival epithelium by hypotonicity. Exp Eye Res.

[12] Carter EP, Matthay MA, Farinas J, Verkman AS (1996). Transalveolar osmotic and diffusional water permeability in intact mouse lung measured by a novel surface fluorescence method. J Gen Physiol.

[13] Cass A, Finkelstein A (1967). Water permeability of thin lipid membranes. J Gen Physiol.

[14] Cesar KR, Magaldi AJ (1999). Thiazide induces water absorption in the inner medullary collecting duct of normal and Brattleboro rats. Am J Physiol.

[15] Dobbs LG, Gonzalez R, Matthay MA, Carter EP, Allen L, Verkman AS (1998). Highly water-permeable type I alveolar epithelial cells confer high water permeability between the airspace and vasculature in rat lung. Proc Natl Acad Sci U S A.

[16] Duvall AJ (1969). The ultrastructure of the external sulcus in the guinea pig cochlear duct. Laryngoscope.

[17] Eckhard A, Gleiser C, Arnold H, Rask-Andersen H, Kumagami H, Müller M, Hirt B, Löwenheim H (2012) Water channel proteins in the inner ear and their link to hearing impairment and deafness. Mol Aspects Med. doi:10.1016/j.mam.2012.06.004 10.1016/j.mam.2012.06.004 22732097

[18] Eckhard A, Gleiser C, Rask-Andersen H, Arnold H, Liu W, Mack A, Muller M, Lowenheim H, Hirt B (2012) Co-localisation of K(ir)4.1 and AQP4 in rat and human cochleae reveals a gap in water channel expression at the transduction sites of endocochlear K(+) recycling routes. Cell and tissue research. doi:10.1007/s00441-012-1456-y 10.1007/s00441-012-1456-y 22802001

[19] Finkelstein A (1987) Water movement through lipid bilayers, pores and plasma membranes: theory and reality. Wiley, New York 10.1126/science.240.4849.228 17800923

[20] Folkesson HG, Matthay MA, Frigeri A, Verkman AS (1996). Transepithelial water permeability in microperfused distal airways. Evidence for channel-mediated water transport. JClin Investig.

[21] Ghosn MG, Tuchin VV, Larin KV (2007). Nondestructive quantification of analyte diffusion in cornea and sclera using optical coherence tomography. Investig Ophthalmol Visual Sci.

[22] Giebel W (1982). The dynamic behavior of inner ear fluids. Laryngol Rhinol Otol.

[23] Gisselsson L (1949). The passage of fluorescein sodium to the labyrinthine fluids. Acta Otolaryngol.

[24] Gresz V, Kwon TH, Hurley PT, Varga G, Zelles T, Nielsen S, Case RM, Steward MC (2001). Identification and localization of aquaporin water channels in human salivary glands. Am J Physiol Gastrointest Liver Physiol.

[25] Guild SR (1927). The circulation of the endolymph. Am J Anat.

[26] Hallpike CS, Cairns H (1938). Observations on the pathology of Meniere's syndrome: (section of otology). Proc Royal Soc Med.

[27] Hamann S, Zeuthen T, La Cour M, Nagelhus EA, Ottersen OP, Agre P, Nielsen S (1998). Aquaporins in complex tissues: distribution of aquaporins 1–5 in human and rat eye. Am J Physiol.

[28] Hansel NN, Sidhaye V, Rafaels NM, Gao L, Gao P, Williams R, Connett JE, Beaty TH, Mathias RA, Wise RA, King LS, Barnes KC (2010). Aquaporin 5 polymorphisms and rate of lung function decline in chronic obstructive pulmonary disease. PloS one.

[29] Hille B (2001). Ion channels in excitable membranes.

[30] Hirt B, Gleiser C, Eckhard A, Mack AF, Müller M, Wolburg H, Löwenheim H (2011). All functional aquaporin-4 isoforms are expressed in the rat cochlea and contribute to the formation of orthogonal arrays of particles. Neuroscience.

[31] Hirt B, Penkova ZH, Eckhard A, Liu W, Rask-Andersen H, Müller M, Löwenheim H (2010). The subcellular distribution of aquaporin 5 in the cochlea reveals a water shunt at the perilymph–endolymph barrier. Neuroscience.

[32] Hofman R, Segenhout JM, Wit HP (2009). Three-dimensional reconstruction of the guinea pig inner ear, comparison of OPFOS and light microscopy, applications of 3D reconstruction. J Microsc.

[33] Huang AJ, Tseng SC, Kenyon KR (1989). Paracellular permeability of corneal and conjunctival epithelia. Investig Ophthalmol Visual Sci.

[34] Ishibashi K, Kuwahara M, Gu Y, Kageyama Y, Tohsaka A, Suzuki F, Marumo F, Sasaki S (1997). Cloning and functional expression of a new water channel abundantly expressed in the testis permeable to water, glycerol, and urea. J Biol Chem.

[35] Jagger DJ, Forge A (2012). The enigmatic root cell—emerging roles contributing to fluid homeostasis within the cochlear outer sulcus. Hear Res.

[36] Jahnke K (1975). The fine structure of freeze-fractured intercellular junctions in the guinea pig inner ear. Acta Otolaryngol Suppl.

[37] Jahnke K (1980). Permeability barriers of the inner ear. Fine structure and function. Fortschritte der Medizin.

[38] Kanoh N, Yagi N, Omura M, Makimoto K (1981). Effects of glycerol on sodium and potassium concentrations in guinea pig perilymph. Arch Otorhinolaryngol.

[39] Karasawa K, Tanaka A, Jung K, Matsuda A, Okamoto N, Oida K, Ohmori K, Matsuda H (2011). Patterns of aquaporin expression in the canine eye. Vet J.

[40] Kimelberg HK (2004). Water homeostasis in the brain: basic concepts. Neuroscience.

[41] Kimura R, Schuknecht HF (1965) Membranous hydrops in the inner ear of the guinea pig after obliteration of the endolymphatic sac. Pract Otorhinolaryngol 27(343–354)

[42] King LS, Kozono D, Agre P (2004). From structure to disease: the evolving tale of aquaporin biology. Nat Rev Mol Cell Biol.

[43] Konishi T, Hamrick PE (1978). Ion transport in the cochlea of guinea pig II. Chloride transport. Acta Otolaryngol.

[44] Konishi T, Hamrick PE, Mori H (1984). Water permeability of the endolymph–perilymph barrier in the guinea pig cochlea. Hear Res.

[45] Konishi T, Hamrick PE, Walsh PJ (1978). Ion transport in guinea pig cochlea I. Potassium and sodium transport. Acta Otolaryngol.

[46] Konishi T, Salt AN, Hamrick PE (1979). Effects of exposure to noise on ion movement in guinea pig cochlea. Hear Res.

[47] Konishi T, Salt AN, Hamrick PE (1981). Effects of hypothermia on ionic movement in the guinea pig cochlea. Hear Res.

[48] Krane CM, Melvin JE, Nguyen HV, Richardson L, Towne JE, Doetschman T, Menon AG (2001). Salivary acinar cells from aquaporin 5-deficient mice have decreased membrane water permeability and altered cell volume regulation. J Biol Chem.

[49] Kuwahara M, Berry CA, Verkman AS (1988). Rapid development of vasopressin-induced hydroosmosis in kidney collecting tubules measured by a new fluorescence technique. Biophys J.

[50] Kuwahara M, Verkman AS (1988). Direct fluorescence measurement of diffusional water permeability in the vasopressin-sensitive kidney collecting tubule. Biophys J.

[51] Lang F, Vallon V, Knipper M, Wangemann P (2007). Functional significance of channels and transporters expressed in the inner ear and kidney. Am J Physiol Cell Physiol.

[52] Lavelle JP, Negrete HO, Poland PA, Kinlough CL, Meyers SD, Hughey RP, Zeidel ML (1997). Low permeabilities of MDCK cell monolayers: a model barrier epithelium. Am J Physiol.

[53] Lawrence M, Wolsk D, Litton WB (1961). Circulation of the inner ear fluids. Ann Otol Rhinol Laryngol.

[54] Lee JH, Chiba T, Marcus DC (2001). P2X2 receptor mediates stimulation of parasensory cation absorption by cochlear outer sulcus cells and vestibular transitional cells. J Neurosci: Off J Soc Neurosci.

[55] Levin MH, Verkman AS (2004). Aquaporin-dependent water permeation at the mouse ocular surface: in vivo microfluorimetric measurements in cornea and conjunctiva. Investig Ophthalmol Visual Sci.

[56] Levine SD, Jacoby M, Finkelstein A (1984). The water permeability of toad urinary bladder. II. The value of Pf/Pd(w) for the antidiuretic hormone-induced water permeation pathway. J Gen Physiol.

[57] Lundquist PG, Kimura R, Wersaell J (1964) Experiments in endolymph circulation. Acta Otolaryngol Suppl 188:SUPPL 188:198+ 10.3109/00016486409134562 14146674

[58] Ma T, Fukuda N, Song Y, Matthay MA, Verkman AS (2000). Lung fluid transport in aquaporin-5 knockout mice. J Clin Investig.

[59] Ma T, Song Y, Gillespie A, Carlson EJ, Epstein CJ, Verkman AS (1999). Defective secretion of saliva in transgenic mice lacking aquaporin-5 water channels. J Biol Chem.

[60] Matsuzaki T, Suzuki T, Koyama H, Tanaka S, Takata K (1999). Aquaporin-5 (AQP5), a water channel protein, in the rat salivary and lacrimal glands: immunolocalization and effect of secretory stimulation. Cell Tissue Res.

[61] Morgan T, Berliner RW (1968). Permeability of the loop of Henle, vasa recta, and collecting duct to water, urea, and sodium. Am J Physiol.

[62] Naftalin L, Harrison MS (1958). Circulation of labyrinthine fluids. J Laryngol Otol.

[63] Naito T (1959) Clinical and pathological studies in Meniere's disease. 60th Annu Meet ORL Soc Jpn (Tokyo)

[64] Nielsen S, Smith BL, Christensen EI, Knepper MA, Agre P (1993). CHIP28 water channels are localized in constitutively water-permeable segments of the nephron. J Cell Biol.

[65] Ning Y, Ying B, Han S, Wang B, Wang X, Wen F (2008). Polymorphisms of aquaporin5 gene in chronic obstructive pulmonary disease in a Chinese population. Swiss Med Wkly.

[66] Nishio N, Teranishi M, Uchida Y, Sugiura S, Ando F, Shimokata H, Sone M, Otake H, Kato K, Yoshida T, Tagaya M, Hibi T, Nakashima T (2013). Polymorphisms in genes encoding aquaporins 4 and 5 and estrogen receptor alpha in patients with Meniere's disease and sudden sensorineural hearing loss. Life Sci.

[67] Oen H, Cheng P, Turner HC, Alvarez LJ, Candia OA (2006). Identification and localization of aquaporin 5 in the mammalian conjunctival epithelium. Exp Eye Res.

[68] Okuno T, Sando I (1987). Localization, frequency, and severity of endolymphatic hydrops and the pathology of the labyrinthine membrane in Meniere's disease. Ann Otol Rhinol Laryngol.

[69] Pallone TL, Kishore BK, Nielsen S, Agre P, Knepper MA (1997). Evidence that aquaporin-1 mediates NaCl-induced water flux across descending vasa recta. Am J Physiol.

[70] Parisi M, Candia O, Alvarez L (1980). Water permeability of the toad corneal epithelium: the effects of pH and amphotericin B. Pflugers Archiv: Eur J Physiol.

[71] Patil RV, Saito I, Yang X, Wax MB (1997). Expression of aquaporins in the rat ocular tissue. Exp Eye Res.

[72] Pender DJ (2013) The nature of Meniere's disease—a lesion analysis. Paper presented at the 13th Triennial Meeting of the International Otopathology Society (Schuknecht-Society), Boston, USA, June 9–11

[73] Preston GM, Agre P (1991). Isolation of the cDNA for erythrocyte integral membrane protein of 28 kilodaltons: member of an ancient channel family. Proc Natl Acad Sci U S A.

[74] Roudier N, Verbavatz JM, Maurel C, Ripoche P, Tacnet F (1998). Evidence for the presence of aquaporin-3 in human red blood cells. J Biol Chem.

[75] Rudert H (1969). Investigation on resorption of the endolymph in the inner ear of the guinea pig. I. Microscopic examinations after injection of trypan blue into the cochlear duct. Arch Klin Exp Ohren Nasen Kehlkopfheilkd.

[76] Salt AN (2004). Acute endolymphatic hydrops generated by exposure of the ear to nontraumatic low-frequency tones. J Assoc Res Otolaryngol.

[77] Salt AN, DeMott J (1997). Longitudinal endolymph flow associated with acute volume increase in the guinea pig cochlea. Hear Res.

[78] Salt AN, DeMott JE (1995). Endolymph volume changes during osmotic dehydration measured by two marker techniques. Hear Res.

[79] Salt AN, Ma Y (2001). Quantification of solute entry into cochlear perilymph through the round window membrane. Hear Res.

[80] Salt AN, Thalmann R, Nadol J (1989). Rate of longitudinal flow of cochlear endolymph. Meniere's disease.

[81] Schnermann J, Chou CL, Ma T, Traynor T, Knepper MA, Verkman AS (1998). Defective proximal tubular fluid reabsorption in transgenic aquaporin-1 null mice. Proc Natl Acad Sci U S A.

[82] Schuknecht HF, Richter E (1980). Apical lesions of the cochlea in idiopathic endolymphatic hydrops and other disorders: pathophysiological implications ORL. J Otorhinolaryngol Relat Spec.

[83] Seo Y, Takamata A, Ogino T, Morita H, Nakamura S, Murakami M (2002). Water permeability of capillaries in the subfornical organ of rats determined by Gd-DTPA(2-) enhanced 1H magnetic resonance imaging. J Physiol.

[84] Shambaugh GE (1908). On the structure and function of the epithelium in the sulcus spiralis externus. Arch Otol.

[85] Shinomori Y, Spack DS, Jones DD, Kimura RS (2001). Volumetric and dimensional analysis of the guinea pig inner ear. Ann Otol Rhinol Laryngol.

[86] Sohara E, Rai T, Miyazaki J, Verkman AS, Sasaki S, Uchida S (2005). Defective water and glycerol transport in the proximal tubules of AQP7 knockout mice. Am J Physiol Renal Physiol.

[87] Song Y, Verkman AS (2001). Aquaporin-5 dependent fluid secretion in airway submucosal glands. J Biol Chem.

[88] Steel KP (1999). Perspectives: biomedicine. The benefits of recycling.. Science.

[89] Steward MC, Garson MJ (1985). Water permeability of *Necturus* gallbladder epithelial cell membranes measured by nuclear magnetic resonance. J Membr Biol.

[90] Steward MC, Seo Y, Rawlings JM, Case RM (1990). Water permeability of acinar cell membranes in the isolated perfused rabbit mandibular salivary gland. J Physiol.

[91] Thorne M, Salt AN, DeMott JE, Henson MM, Henson OW, Gewalt SL (1999). Cochlear fluid space dimensions for six species derived from reconstructions of three-dimensional magnetic resonance images. Laryngoscope.

[92] van Os CH, Slegers JF (1973). Path of osmotic water flow through rabbit gall bladder epithelium. Biochim Biophys Acta.

[93] Verkman A (1999). Water permeation across membranes. In: Membrane permeability—100 years since Ernest Overton.

[94] Verkman AS (2000). Water permeability measurement in living cells and complex tissues. J Membr Biol.

[95] Verkman AS (2012). Aquaporins in clinical medicine. Annu Rev Med.

[96] Verkman AS, Yang B, Song Y, Manley GT, Ma T (2000) Role of water channels in fluid transport studied by phenotype analysis of aquaporin knockout mice. Exp Physiol, 85 Spec No:233S-241S 10.1111/j.1469-445x.2000.tb00028.x 10795927

[97] Wang KS, Ma T, Filiz F, Verkman AS, Bastidas JA (2000). Colon water transport in transgenic mice lacking aquaporin-4 water channels. Am J Physiol Gastrointest Liver Physiol.

[98] Yamakawa K (1938). Über die pathologische Veränderung bei einem Menière-Kranken. J Otorhinolaryngol Soc Jpn.

[99] Yan D, Zhu Y, Walsh T, Xie D, Yuan H, Sirmaci A, Fujikawa T, Wong AC, Loh TL, Du L, Grati M, Vlajkovic SM, Blanton S, Ryan AF, Chen ZY, Thorne PR, Kachar B, Tekin M, Zhao HB, Housley GD, King MC, Liu XZ (2013). Mutation of the ATP-gated P2X(2) receptor leads to progressive hearing loss and increased susceptibility to noise. Proc Natl Acad Sci U S A.

[100] Yang B, Verkman AS (1997). Water and glycerol permeabilities of aquaporins 1–5 and MIP determined quantitatively by expression of epitope-tagged constructs in *Xenopus* oocytes. J Biol Chem.

[101] Zelenina M, Brismar H (2000). Osmotic water permeability measurements using confocal laser scanning microscopy. Eur Biophys J.

[102] Zidanic M, Brownell WE (1990). Fine structure of the intracochlear potential field I. The silent current. Biophys J.

